# Bacterial Pathogens and Community Composition in Advanced Sewage Treatment Systems Revealed by Metagenomics Analysis Based on High-Throughput Sequencing

**DOI:** 10.1371/journal.pone.0125549

**Published:** 2015-05-04

**Authors:** Xin Lu, Xu-Xiang Zhang, Zhu Wang, Kailong Huang, Yuan Wang, Weigang Liang, Yunfei Tan, Bo Liu, Junying Tang

**Affiliations:** 1 State Key Laboratory of Pollution Control and Resource Reuse, School of the Environment, Nanjing University, Nanjing, China; 2 Environmental Science Research Institute of Jiangsu, Nanjing, China; 3 Zhengzhou Sewage Purification Company, Zhengzhou, China; 4 Research Institute of Nanjing University at Lianyungang, Lianyungang, China; University of British Columbia, CANADA

## Abstract

This study used 454 pyrosequencing, Illumina high-throughput sequencing and metagenomic analysis to investigate bacterial pathogens and their potential virulence in a sewage treatment plant (STP) applying both conventional and advanced treatment processes. Pyrosequencing and Illumina sequencing consistently demonstrated that *Arcobacter* genus occupied over 43.42% of total abundance of potential pathogens in the STP. At species level, potential pathogens *Arcobacter butzleri*, *Aeromonas hydrophila* and *Klebsiella pneumonia* dominated in raw sewage, which was also confirmed by quantitative real time PCR. Illumina sequencing also revealed prevalence of various types of pathogenicity islands and virulence proteins in the STP. Most of the potential pathogens and virulence factors were eliminated in the STP, and the removal efficiency mainly depended on oxidation ditch. Compared with sand filtration, magnetic resin seemed to have higher removals in most of the potential pathogens and virulence factors. However, presence of the residual *A*. *butzleri* in the final effluent still deserves more concerns. The findings indicate that sewage acts as an important source of environmental pathogens, but STPs can effectively control their spread in the environment. Joint use of the high-throughput sequencing technologies is considered a reliable method for deep and comprehensive overview of environmental bacterial virulence.

## Introduction

Sewage is considered as a habitat for various human bacterial pathogens, and discharge of sewage into environmental water may threaten public health [1–3]. Bacterial pathogenesis often depends on specific virulence factors (VFs) [3], which enable microorganisms to proliferate and cause tissue damage or systemic inflammation in a host niche [4]. In China, sewage treatment plants (STPs) normally consist of primary sedimentation and secondary biological treatment [5, 6]. Because of the specific effects of light, temperature and oxygen availability on shaping bacterial community structure, the primary sedimentation and conventional secondary biological treatment could remove about 30% and 80% pathogens from influent, respectively [7, 8]. Despite of high removal of pathogens in STPs [9, 10], the residual pathogens in effluent still deserve public concerns. It has been indicated that STPs are important sources for environmental pathogens [11], such as *Legionella pneumophila* breaking out from Norway STPs [12]. In order to reduce the potential health risk induced by pathogens, disinfection methods (e.g. chlorination) are usually used in sewage treatment [13], although the disinfection techniques are known capable of inducing adverse effects, such as disinfection by-products generation [14] and antibiotic resistance enhancement [15]. However, few studies have been conducted to investigate the fates of bacterial pathogens in advanced sewage treatment systems.

Human pathogens can survive and grow in STPs [2], but they are minor components of bacterial community in sewage and have to be enriched for detection [3]. Traditionally, total coliforms and fecal coliforms are used to indicate the contamination of water or wastewater by pathogens [16]. However, most of the environmental bacteria are unculturable [17], and the bacteria culture may cause bias in detection of most pathogens. Recently, molecular technologies, such as polymerase chain reaction (PCR) [18], quantitative real time PCR (q-PCR) [19, 20] and microarray [20], have been widely used to detect pathogens in sewage and natural waters. Nevertheless, these technologies can only specifically detect certain pathogens and cannot provide a comprehensive insight of potential pathogens in the environment. High-throughput sequencing, a promising technology, can provide enough sequencing depth to cover the complex microbial community of the environment [21]. Recent studies have investigated the diversity of pathogens in activated sludge of STPs by using 454 pyrosequencing of 16S rRNA gene amplicons [22], or Illunima high-throughput sequencing of environmental genomes [23, 24].

This study aimed to investigate the fates of pathogens and VFs in both conventional and advanced treatment processes of a full-scale STP by joint use of 454 pyrosequencing, Illumina high-throughput sequencing and q-PCR. This study provided a comprehensive insight into bacterial pathogens and community composition in the STPs and demonstrated the applicability of the high-throughput sequencing technologies in environmental pathogens detection.

## Materials and Methods

### Sampling and DNA extraction

Wastewater and sludge samples were collected from Wulongkou STP (Zhengzhou City, Henan Province, China; 34.78 deg N, 113.61 deg E) with daily treatment of 100,000 m^3^ of sewage. S1 Table shows detailed information about the operational processes and wastewater quality. We would like to state that the plant have approved this study which did not involve endangered or protected species. In the STP, sewage underwent a series of conventional treatment processes including grating, grit filtration and activated sludge treatment and secondary settling. The effluent from secondary settling tank was separately subject to two advanced treatment processes of coagulation/precipitation and resin filtration (S1 Fig). Samples were simultaneously collected from six locations along wastewater flow, including sewage influent (SI), primary effluent (PE), activated sludge (AS), secondary effluent (SE), final sand filter effluent (FFE) and final resin effluent (FRE) at three time points of November 2012, February 2013 and May 2013 (S1 Fig, S2 Table).

Activated sludge samples (about 50 ml each) were centrifuged at 4,000 rpm for 10 min to collect approximately 200 mg of the pellet for DNA extraction. The influent and effluent samples were filtered through 0.45-μm micropore membrane with diameter of 50 mm (Xinya Co., China) to collect cell pellets for DNA extraction. For each sample, the extraction was conducted in duplicate using the FastDNA Soil Kit (MP Biomedicals, CA, USA). The DNA concentration and purity were determined by microspectrophotometry (NanoDropND-2000, NanoDrop Technologies, Wilmington, DE). The volume of the raw and treated sewage filtered through membrane was recorded to calculate the amount of DNA in given volume of sewage water.

### 454 Pyrosequencing and Illumina high-throughput sequencing

To avoid temporal variation, equal mass of DNA extracted from the three samples collected from each location at different time points were mixed for pyrosequencing on Roche 454 FLX Titanium platform (Roche, USA) and high-throughput sequencing on Illumina Hiseq 2000 (Illumina, USA). The extracted DNA was sent to Beijing Genome Institute (BGI, China) for Illumina shotgun sequencing. The sequencing strategy was “Index 101 PE” (Paired End sequencing, 101-bp reads and 8-bp index sequence), which generated nearly equal amount of clean reads for each sample. The raw reads containing three or more “N” or contaminated by adapter (> 15 bp overlap) were removed, and the filtered clean reads were used for metagenomic analyses.

The bacterial 16S rRNA gene was amplified with a set of primers targeting the hypervariable V3-V4 regions (about 460 bp). The primers used were V3F (5'- ACTCCTACGGGAGGCAGCAG-3') and V4R (5'- TACNVGGGTATCTAATCC-3'). PCRs were conducted in 50 μL reaction volume, containing 1 × Pfx Amplification Buffer (Invitrogen, USA), 0.4 mM dNTP, 2 mM MgSO_4_, 0.4 μM each fusion primer, 1 μL of template DNA and 2 U of Platinum Pfx DNA Polymerase (Invitrogen, USA). PCR protocol included initial denaturation at 94°C for 3 min, followed by 30 cycles of 94°C for 30 s, annealing at 62°C for 30 s and extension at 70°C for 45 s, with a final elongation step at 70°C for 7 min. A 10-nucleotide “barcode” was added into forward primer to separate the corresponding reads from the data pool generated in a single pyrosequencing run. To minimize the impact of potential early round PCR errors, PCR amplicon libraries were prepared by combining three independent PCR products for each sample. The PCR products were purified by MiniBest DNA Fragment Purification Kit Ver.4.0 (TaKaRa, Japan). Purified PCR products were mixed and sent to Majorbio Co. Ltd. (Shanghai, China) for pyrosequencing. Average length of the pyrosequencing reads generated was about 494 bp, and the detailed sequencing information is shown in S3 Table. The pyrosequencing and Illumina sequencing data have been deposited into the NCBI Short-reads Archive Database and MG-RAST server, respectively, under the accession numbers listed in S2 Table.

### Bioinformatics analysis on Illumina high-throughput sequencing

The bioinformatics strategy of the Illumina data included four steps: (i) quality filtering and normalization, (ii) alignment, (iii) annotation, and (iv) statistics analysis. For quality control, the sequences were denoised by FASTX toolkit tools in GALAXY using parameters (quality cut-off value 30; percent of bases cut-off value 75) [25]. In order to compare the six samples at the same sequencing depth, the number of the filtered reads from each sample was normalized to 9,000,000 by a self-written Python script.

To identify the 16S rRNA gene, sequences of each dataset were aligned to the Silva database [26] using the local BLASTN tool. The output files were then imported into the MEGAN tool [27] to conduct the taxonomic assignment with the lowest common ancestor (LCA) algorithm using default parameters (min score 50, top percent 10, win score 0.0, and min complexity 0.3) [28]. The synonyms file (silva2ncbi.map) downloaded from MEGAN website (http://ab.inf.unituebingen.de/software/megan/) was used to map Silva accession numbers to NCBI taxa. Each of the dataset was imported into the MetaPhlAn online platform in GALAXY (http://huttenhower.sph.harvard.edu/galaxy) to profile the microbial community using the default parameters [29]. The output files were calculated and collated into a table file to generate a heatmap by R.

To establish a local database of VFs, the protein sequences of pathogenicity islands and virulence proteins with specific pathogenic function were downloaded from MvirDB [1], a microbial database of protein toxins, VFs and antibiotic resistance genes. The Illumina datasets were aligned against the purified MvirDB by off-line BLASTX, and a sequencing read was annotated as one VF (pathogenicity islands or virulence proteins) if its best BLASTX hit had a similarity above 90% over an alignment of more than 50 bp [15]. To compile, confirm and validate the collection of data, each of the MvirDB protein sequences were manually aligned against the NCBI Protein Database (http://blast.ncbi.nlm.nih.gov/) to check whether one protein is specific for the pathogenic hosts, and the ones not directly involving pathogenesis were deleted from the database.

### Bioinformatics analysis on 454 pyrosequencing

Pyrosequencing reads of each data set were denoised using the Mothur platform [30]. Sequences were trimed using the “trim.seqs” command [31], (1) to trim off the adapters, barcodes and primers, (2) to remove the low quality reads and the reads containing ambiguous “N”, and (3) to remove the reads shorter than 300 bp. Subsequently, pyrosequencing errors were removed using the “pre.cluster” command [21], and PCR chimeras were removed using Chimera slayer [21]. For fair comparison, sequence number was normalized to 6,200 for each sample using a self-written Python script.

All the filtered sequences were compared with the Silva database [26] by the local BLASTN tool, and the output sequences were then assigned to NCBI taxonomies with MEGAN [22] to profile the bacterial communities. Sequences identified as archaea and eukaryon were filtered out. To further identify pathogens at species level, the retained sequences were aligned to a human pathogenic bacteria 16S rRNA gene database [23] downloaded from a homepage of the University of Hong Kong (http://web.hku.hk/~zhangt/ZhangT.htm), and pathogenic bacteria were determined if hits similarity was over 97% [32].

### Quantitative real-time PCR

The genes encoding beta-glucuronidase (*uidA*) of *Escherichia coli* [19], extracellular lipase (*lip*) of *Aeromonas hydrophila* [33], gyrase A subunit (*gyrA*) outside the quinolone resistance determining region of *Arcobacter butzleri* [34] and phosphate-regulated porin (*phoE*) of *Klebsiella pneumoniae* [35] were used as specific indicators to quantify the four potential pathogens in the sewage treatment plant, respectively. Specificity of the primer sets for the pathogens was confirmed by online BLAST search (http://blast.ncbi.nlm.nih.gov/Blast.cgi). Abundance of the four genes in the samples was determined by q-PCR on Corbett Real-Time PCR Machine with Rotor-Gene 6000 Series Software 1.7 (QIAGEN, the Netherlands). q-PCR of each gene was conducted in triplicate with the primer sets listed in S4 Table. The recombinant plasmids obtained by molecular cloning of the target genes were used to generate standard curves. In detail, PCRs of target genes were conducted in a 30 μL volume containing 1×PCR buffer, 100 μM dNTP, 2 pmol of each primer, 150 ng of DNA template and 0.8 U of ^EX^Taq polymerase (TaKaRa, Japan). PCR products of target genes were purified using PCR Quick-Spin PCR Product Purification Kit Ver.4.0 (TaKaRa, Japan) and cloned using pMD18-T Vector (TaKaRa, Japan). The sequences of the amplicons were deposited into GenBank under the accession numbers listed in S4 Table. Plasmids carrying target genes were extracted and purified using MiniBest Plasmid Purification Kit (TaKaRa, Japan). Purified plasmid concentrations were determined by microspectrophotometry (NanoDropND-2000, NanoDrop Technologies, Wilmington, DE) [36]. For q-PCR, the 20-μL reaction volume contained 10 μL of SYBR Premix ^EX^Taq Super Mix (TaKaRa, Japan), 8 μL of DNA template, 10 μM each primer and ddH_2_O [37], and the reaction conditions included: initial denaturation at 94° for 3 min, followed by 40 thermal cycles (S4 Table) and final extension at 72°C for 45 s. The specificity of q-PCR products was confirmed by melting curves observation and agarose (1%) gel electrophoresis.

## Results

### Detection of potential pathogens by 454 pyrosequencing

**Fig 1:**
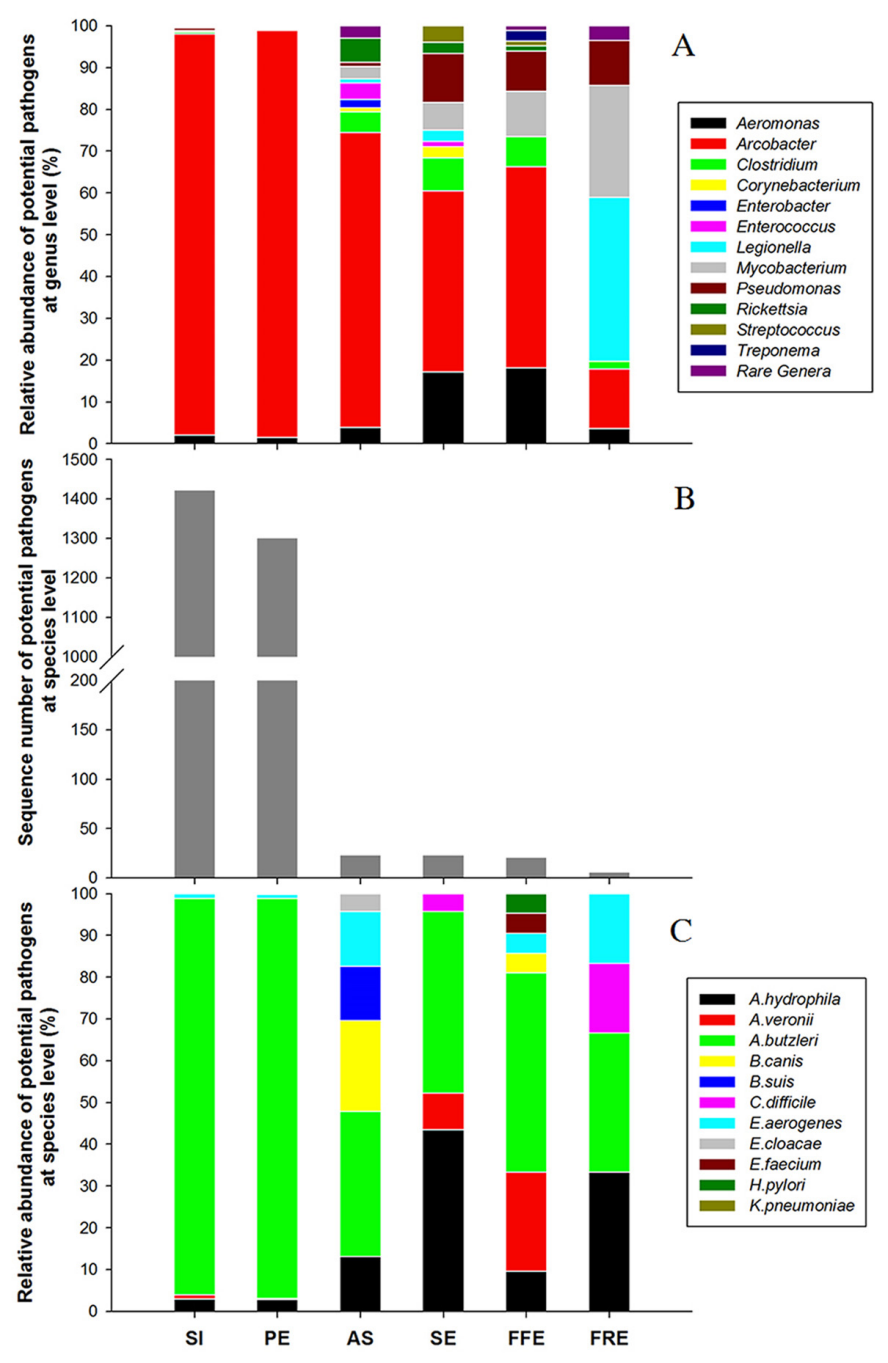
Occurrence and abundance of potential pathogens at genus and species levels revealed by 454 pyrosequencing of 16S rRNA gene amplicons. (A) Relative abundance of potential pathogens at genus level. The effective pyrosequencing reads were classified using MEGAN. Rare genera refer to the taxa with maximum abundance <1% in any sample, including *Bacillus*, *Campylobacter*, *Helicobacter*, *Klebsiella*, *Leptospira*, *Neisseria* and *Serratia*. (B) Sequence number of potential pathogens at species level under the same sequencing depth (6200 reads). (C) Relative abundance of potential pathogens at species level. All the effective sequences identified as potentially pathogenic species were aligned using the local BLASTN tool. The relative abundance was obtained by normalizing the sequence number of each pathogenic taxon to the total number of all pathogenic taxa in one sample.

Pyrosequencing of 16S rRNA gene amplicons revealed that the bacterial community shifted greatly along the treatment processes (S2 Fig). At genus level, the relative number of the sequences assigned to known pathogens was 3,428 (55.33%) for SI, 3,883 (62.63%) for PE, 102 (1.65%) for AS, 76 (1.23%) for SE, 83 (1.34%) for FFE and 56 (0.90%) for FRE (S5 Table), and a total of 20 genera containing potentially pathogenic species were detected in the six samples (Fig 1A). Among the genera identified, *Acrobacter* had the highest abundance in each of SI, PE, AS, SE and FFE, accounting for 43.42%-97.37% of all pathogenic sequences, and the genus also occupied 14.29% in FRE (Fig 1A). At species level, pyrosequencing showed that 1,422 (6 species, 22.95%), 1,302 (7 species, 21.00%), 23 (7 species, 0.37%), 23 (4 species, 0.37%), 21 (7 species, 0.34%) and 6 (4 species, 0.10%) sequences were closely related (with similarity over 97%) to known pathogens in SI, PE, AS, SE, FFE and FRE, respectively (Fig 1B), demonstrating that the sewage treatment processes can effectively reduce the relative abundance of the pathogens. In absolute terms, over 99% of the potential pathogens in SI (2.68×10^4^ sequences per milliliter) were eliminated by the treatment processes (S6 Table). Among the species identified, *Arcobacter butzleri* had the highest abundance in the STP, and the relative abundance ranged from 33.33% to 94.87% of total pathogenic bacteria in the six samples (Fig 1C). Additionally, *Aeromonas hydrophila* was also found prevalent in SE and FRE (Fig 1C).

### Detection of potential pathogens by Illumina sequencing

**Fig 2:**
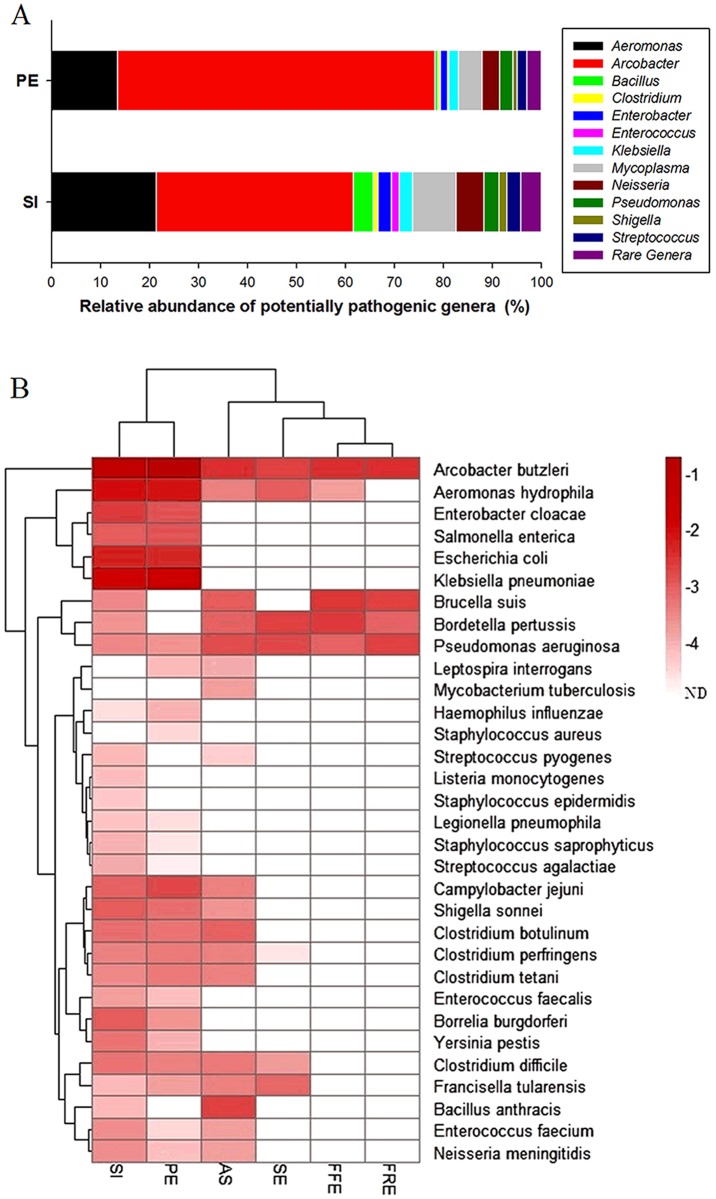
Diversity and relative abundance of potential pathogens at genus (A) and species (B) levels revealed by Illumina shotgun sequencing of the metagenomes. (A) Relative abundance of potentially pathogenic genera. The denoised reads were aligned using MEGAN against 16S rRNA gene Silva database. Rare genera, indicating the taxa with maximum abundance lower than 1% in any sample, include *Bordetella*, *Campylobacter*, *Corynebacterium*, *Escherichia*, *Francisella*, *Haemophilus*, *Helicobacter*, *Legionella*, *Leptospira*, *Listeria*, *Mycobacterium*, *Salmonella*, *Serratia*, *Staphylococcus*, *Treponema*, *Vibrio* and *Yersinia*. (B) Heat map illustrating relative abundance (log) of potential pathogens at species level generated by MetaPhlAn. The relative abundance was obtained by normalizing the sequence number of each pathogenic taxon to the total number of all pathogenic taxa in one sample.

For a further investigation of potential pathogens in the STP, the Illumina sequencing reads assigned to 16S rRNA gene were used to determine the diversity of potential pathogens. Results showed that the potential pathogens in the STP were relatively affiliated with 29 genera in each of SI (6,952 sequences) and PE (10,086 sequences), among which *Arcobacter* had the highest relative abundance (Fig 2A). Both the conventional and advanced sewage treatment processes applied in the STP demonstrated high pathogen removal efficiency since only 440, 418, 292 and 219 sequences were found closely related to known pathogens in AS, SE, FFE and FRE, respectively (S7 Table). Comparison in absolute terms showed the similar results (S6 Table). Further analysis of the Illumina sequencing datasets by MetaPhlAn showed that a total of 32 species of the potential pathogens were detected in the six samples (Fig 2B), among which 30, 25, 18, 7, 5 and 4 species were present in SI, PE, AS, SE, FFE and FRE, respectively. Among the species, *A*. *butzleri* was most abundant in each sample. *A*. *hydrophila*, *E*. *coli* and *K*. *pneumoniae* were also found prevalent in SI and PE (over 1.85% each). *A*. *hydrophila*, *Bordetella pertussis* and *Pseudomonas aeruginosa* also had high abundance in SE after oxidation ditch treatment. Comparatively, *A*. *hydrophila* had a decreased abundance and *Brucella suis* had an increased abundance in both FFE and FRE. *Bordetella pertussis* in FFE and *Pseudomonas aeruginosa* in FRE were also found to be the dominant potential pathogens.

### Removal of four pathogens quantified by q-PCR

**Fig 3:**
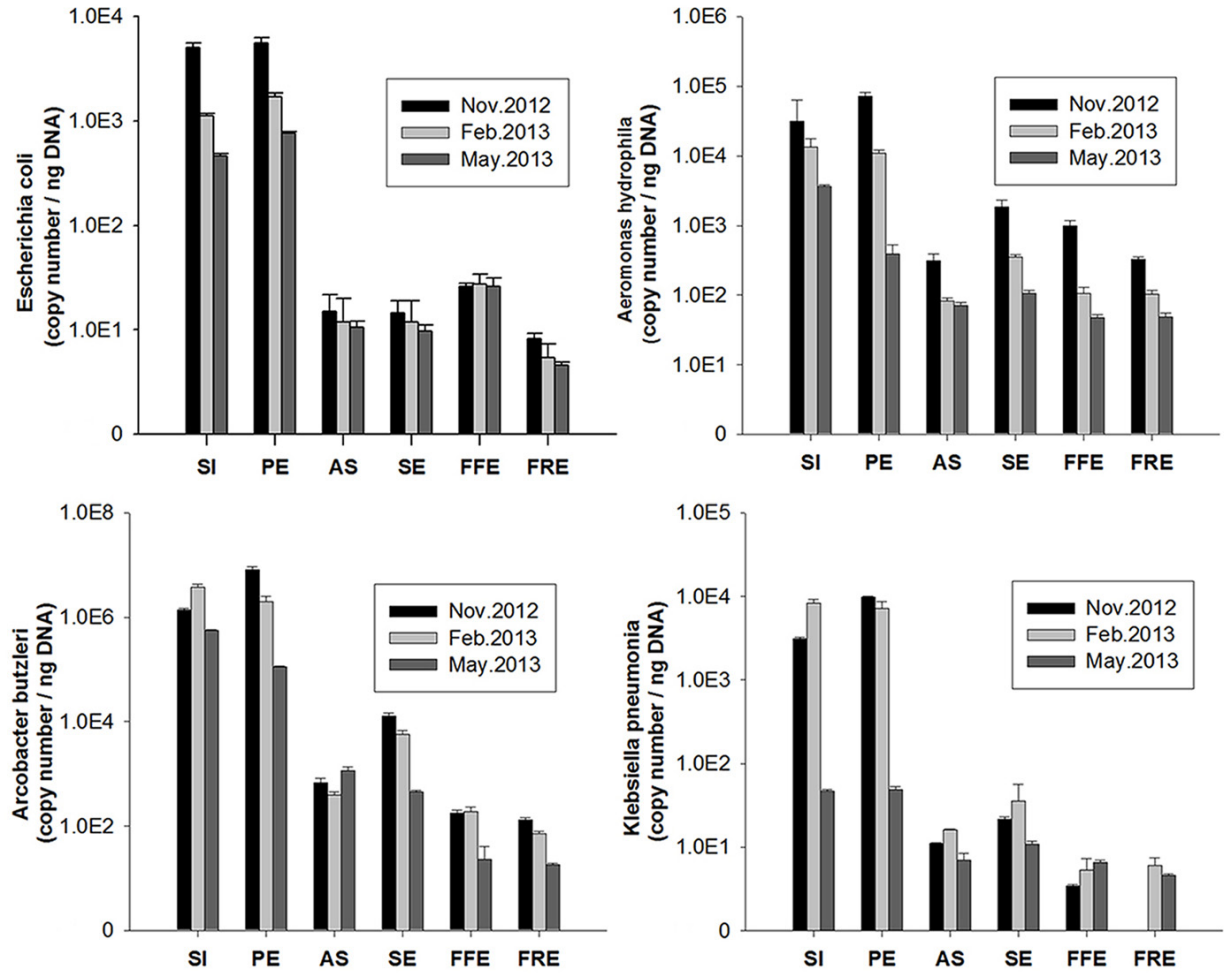
Abundance of four potentially pathogenic bacteria in the samples collected from the STP at different time points. The abundance was determined by quantitative real time PCR and normalized to one nanogram of extracted DNA.

To confirm the metagenomic results, q-PCR was used to investigate the removals of four potential pathogens in relative and absolute terms by different sewage treatment processes (Fig 3 and S4 Fig). Results consistently showed that *A*. *butzleri*, among the four pathogens, had the highest abundance in each sampling location and time point, followed by *A*. *hydrophila*, *E*. *coli* and *K*. *pneumonia*, which agrees with the results of the high-throughput sequencing technologies. Overall, about 99% of pathogens in the raw sewage were finally removed in the STP, and comparison of the different treatment processes used in the STP showed that the removal effectiveness mainly depended on the oxidation ditch. Magnetic resin processes were found to remove 99% of *E*. *coli*, but sand filter tank seemed to accumulate the pathogen. Magnetic resin also showed higher removals in *A*. *hydrophila* and *K*. *pneumonia* than sand filtration (*p* <0.05 each), which is consistent with the results of metagenomic analyses.

### Detection of VFs by Illumina high-throughput sequencing

**Fig 4:**
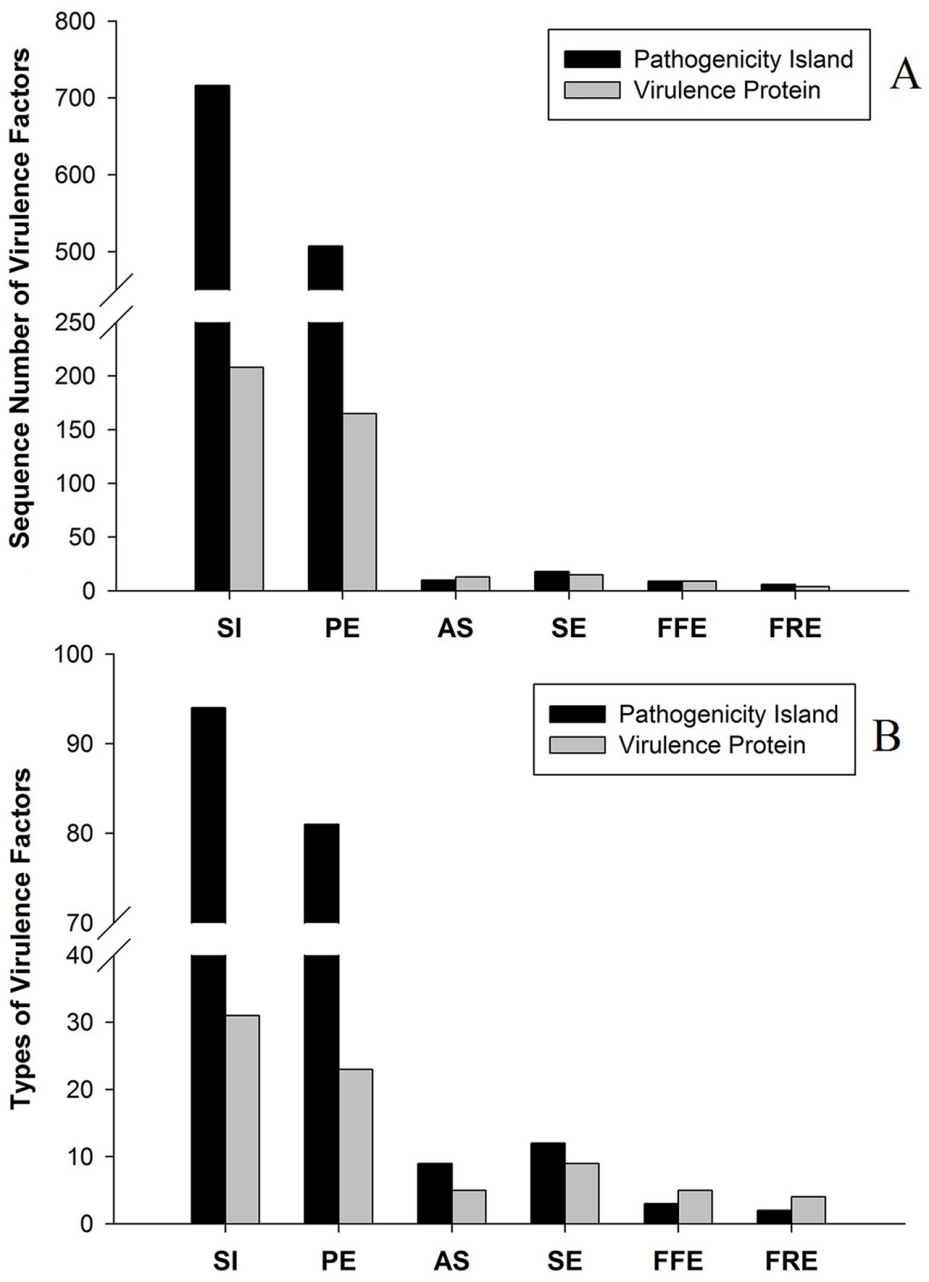
Metagenomic analyses of virulence factors (VFs) at six locations along sewage flow in the STP. Sequence number (A) and types (B) of virulence factors were calculated based on alignment of the Illumina shotgun sequences (normalized to 9,000,000 reads for each sample) against MvirDB protein database.

BLAST against MvirDB protein database showed that 716 sequences from SI, 507 sequences from PE, 10 sequences from AS, 18 sequences from SE, 9 sequences from FFE and 6 sequences from FRE were annotated as the known pathogenicity islands, which were assigned to 94, 81, 9, 12, 3 and 2 types, respectively. A total of 208 sequences from SI, 165 sequences from PE, 13 sequences from AS, 15 sequences from SE, 9 sequences from FFE and 4 sequences from FRE were assigned to 31, 23, 5, 9, 5 and 4 types of known virulence proteins, respectively. Similar to pathogens, both pathogenicity islands and virulence proteins in the STP were mainly removed by oxidation ditch (Fig 4), which agrees with the results in absolute terms (S6 Table).

## Discussion

In this study, potential pathogens in the STP were detected by joint use of 454 pyrosequencing and Illumina high-throughput sequencing, two promising technologies reliable for quantification of genetic diversity within environmental communities [38]. All sequences generated by the two technologies were blasted against Silva database and then imported into MEGAN using the same LCA algorithm parameters. LCA has been proved more accurate than Best Hit for annotating short sequences at genus or species level [23]. It has also been indicated that MEGAN can provide more reliable taxonomy results than RDP classifier in 454 pyrosequencing data process [22] and can assign sequences to species deposited at NCBI [27], so we used MEGAN for metagenomic analyses to detect the potential pathogens in the STP. Moreover, MetaPhlAn, a more reliable tool to profile environmental communities using the database of clade-specific marker genes [5, 23], was applied to determine pathogens based on Illumina sequencing.

This study showed that Illumina sequencing portrayed a much more complex pathogenic community than 454 pyrosequencing. Due to bias from PCRs of 16S rRNA gene, pyrosequencing only identified 11 species of potentially pathogenic bacteria, overlooking some minor populations. The PCR bias can be avoided by direct shotgun sequencing of environmental genomes, so Illumina sequencing revealed more species of the potential pathogens in the STP [23].

Despite of the difference mentioned above, pyrosequencing and Illumina sequencing provided consistent results for detection of the major genera (e.g. *Arcobacter*) and species of bacterial pathogens (e.g. *A*. *butzleri*, *A*. *hydrophila* and *K*. *pneumonia*) in the STP, which was further supported by q-PCR. Moreover, the three technologies consistently indicated that *A*. *butzleri* was the most abundant pathogenic species in the STP. *A*. *butzleri*, a free-living and waterborne pathogen, can cause gastrointestinal diseases such as diarrhea [39]. Previous studies have revealed that *Arcobacter* genus dominates in influent sewage of different STPs based on pyrosequencing of 16S rRNA gene [21]. Within the genus, *A*. *butzleri* usually has highest abundance in sewage, and presence of *Arcobacter* sp. in environmental waters indicates high levels of fecal pollution [40]. The pathogens *A*. *hydrophila*, *K*. *pneumoniae* and *E*. *coli* were also frequently detected in sewage by using molecular methods [22] or culture-dependent methods [41, 42].

VFs responsible for pathogenesis were further analyzed by Illumina sequencing and metagenomic analyses. This study revealed occurrence of various types of pathogenicity islands and virulence proteins in the STP. Pathogenicity islands, a distinct class of genomic islands acquired by horizontal gene transfer, are often incorporated in the genome of pathogenic organisms, but absent in nonpathogenic ones [43]. Some mobile genetic elements (e.g. transposon, integron, plasmids etc.), which are normally capable of gene transfer, can directly encode pathogenicity factors [44]. Virulence proteins, often acquired by horizontal genetic transfer [45], can facilitate bacterial pathogenesis by interfering with host cell signal transduction and other cellular processes [46]. Although types and abundances of virulence factors decreased dramatically after the sewage treatment, several ones with serious pathogenicity and virulence were still present in the final effluent (S8 and S9 Tables). For example, STM1008, a gifsy-2 prophage, can induce *Salmonella*’s ability to establish a systemic infection in mice [47]. Heat shock proteins (Hsp70), which are normally generated in response to adverse environmental conditions, were reported to contribute to virulence as adhesins for invading the host cell or in signaling the immune system [48].

Pyrosequencing and Illumina sequencing consistently showed that the abundance and diversity of the potential pathogens and VFs obviously declined along water stream of the sewage treatment systems. This changing trend was subsequently confirmed by the results of q-PCR. Oxidation ditch was found highly effective in removing the pathogens and VFs, which may result from the microbial structure shift from raw sewage to sludge and treated sewage. The removal of suspended solids by oxidation ditch may also contribute to the decrease in the quantity of the potential pathogens and VFs in per milliliter of sewage water. Previous studies have also revealed that aerobic treatment can contribute to the removal of pathogens and VFs in sewage based on molecular detection or bacterial culture. By microarray and q-PCR, Lee et al. [20] revealed that activated sludge process had removal efficiency of over 99% for each of 12 tested pathogens including *A*. *hydrophila*, *K*. *pneumoniae* and *E*. *coli*. Membrane filtration culture of pathogens also demonstrated that activated sludge process can remove nearly all cells of *A*. *hydrophila* [49]. By q-PCR, our previous studies have also revealed that integrases and transposases genes can be effectively eliminated from sewage by aerobic treatment [50, 51]. The possible explanation is that growth of the pathogenic bacteria dominating in anaerobic or anoxic environments of raw sewage was inhibited when the sewage entered in the aerobic tank.

Comparison between the two advanced sewage treatment processes indicated that magnetic resin treatment had higher removals in most of the potential pathogens and VFs than sand filtration. Little information is available about the effectiveness of the two processes in removing pathogens or VFs. It is known that magnetic resin process can effectively remove hydrophilic organic compounds [52] and heavy metals [53] from biologically treated secondary effluent within a short contact time. Additionally, magnetic resin can increase disinfection capability of ozone by reducing the ozone decay rates [54], but no studies have been conducted to investigate the effect of magnetic resin on pathogen removal. Its removal effect on pathogen may mainly result from the reinforced capability of magnetic resin in adsorbing and accumulating residuum of organisms from treated secondary effluent [52]. Although most of the pathogens and virulence factors were eliminated in the STP, presence of the opportunistic pathogens (e.g. *A*. *butzleri*) in the effluent still deserves research concerns, as little information is available on their environmental fates in receiving water bodies.

To our knowledge, this is the first study jointly using the two different high-throughput sequencing technologies to investigate bacterial virulence in STPs. Technical complementation of the two sequencing methods and q-PCR can provide a more comprehensive and accurate insight into environmental bacterial virulence. However, compared with culture-based methods, the molecular technologies still have some limitations on specificity, since pathogenicity may vary among the different strains within one species [22]. For example, adhesiveness, invasiveness, cytotoxicity and diarrhea infectivity of *A*. *butzleri* strains varied among different animal hosts [39].

In conclusion, joint use of Illumina high-throughput sequencing, 454 pyrosequencing and q-PCR reveals that the STPs are important environmental reservoirs of various potential pathogens, among which *Arcobacter* are prevalent. *A*. *butzleri*, *A*. *hydrophila* and *K*. *pneumonia* are the major species of potential pathogens dominating in raw sewage. Illumina sequencing also reveals occurrence of various types of pathogenicity islands and virulence proteins in the STPs. Most of the potential pathogens and VFs can be eliminated in the STPs applying conventional and advanced treatment processes, and the removal efficiency mainly depends on aerobic treatment. As a novel advanced treatment process, magnetic resin shows higher removals in most of the potential pathogens and VFs than sand filtration. However, *A*. *butzleri* can still persist in the final effluent, which deserves more concerns.

## Supporting Information

S1 Table Information of operational processes and wastewater quality of the sewage treatment plant. (DOCX)

S2 Table Information of the water/sludge sampling from the sewage treatment plant and accession numbers of the sequencing datasets deposited into publicly available databases. (DOCX)

S3 Table Sequencing information of Illumina high-throughput sequencing and 454 pyrosequencing data analyzed. (DOCX)

S4 Table Primer sequences and conditions for q-PCR of the target genes indicating human bacterial pathogens. (DOCX)

S5 Table Numbers of the sequences assigned to genera containing potentially pathogenic species obtained from annotation of 454 pyrosequencing reads by using MEGAN. (DOCX)

S6 Table Absolute abundance of genera containing potentially pathogenic species and virulence factors detected in sewage samples. (DOCX)

S7 Table Numbers of the sequences assigned to genera containing potentially pathogenic species obtained from annotation of Illumina high-throughput sequencing reads by using MEGAN. (DOCX)

S8 Table Pathogenicity islands detected at six locations along sewage flow in the STP by Illumina high-throughput sequencing. (DOCX)

S9 Table Virulence proteins detected at six locations along sewage treatment in the STP by Illumina high-throughput sequencing. (DOCX)

S1 Fig Sampling locations along the sewage treatment processes in Wulongkou STP located in Zhengzhou City, China. (TIF)

S2 Fig Relative abundance of different phyla and classes in *Proteobacteria* at six stages of sewage treatment. (TIF)

S3 Fig Percentages of unclassified pyrosequencing reads at seven taxonomic levels for the six samples. (TIF)

S4 Fig Absolute abundance of four potentially pathogenic bacteria in sewage samples collected from the STP at different time points. (TIF)
