# Supplementary material for: Bacterial Pathogens and Community Composition in Advanced Sewage Treatment Systems Revealed by Metagenomics Analysis Based on High-Throughput Sequencing
Source: PLoS One. 2015 May 4;10(5):e0125549. doi: 10.1371/journal.pone.0125549 (PMC4418606; doi:10.1371/journal.pone.0125549)
Supplement: S1 Table — (DOCX) [file pone.0125549.s001.docx]

**S1 Table.** Information of operational processes and wastewater quality of the sewage treatment plant.

| Operational Parameters | Value | | | | |
| --- | --- | --- | --- | --- | --- |
| Flow rate | 100 000 m^3^d^-1^ | | | | |
| Sludge Load (F/M) | 0.080-0.204 kgCOD/(kgMLSS·d) | | | | |
| Sludge Volume Index (SVI) | 60-121 ml/g | | | | |
| Mixed Liquor Suspended Solids (MLSS) | 2869-4065 mg/L | | | | |
| Temperature (T) | 13.1-27.7 ℃ | | | | |
| pH | 6.72-7.55 | | | | |
| Wastewater Quality (mg/L) | | | | | |
| Water Samples | SI | PE | SE | FFE | FRE |
| Chemical Oxygen Demand (COD) | 275-517 | 218-382 | 17-38 | 12-23 | 10-21 |
| Ammonia Nitrogen (NH_3_-N) | 26.1-34.7 | 25.8-33.9 | 1.57-3.48 | 0.73-2.91 | 0.68-2.82 |
| Total Nitrogen (TN) | 23.9-54.6 | 24.8-56.7 | 5.28-14.10 | 8.62-12.5 | 9.03-12.62 |
| Total Phosphorus (TP) | 5.8-12.6 | 5.4-12.8 | 0.16-0.38 | 0.16-0.42 | 0.18-0.33 |
| Suspended Solids (SS) | 608-954 | 286-423 | 10-21 | 4-9 | 3-8 |
