# Supplementary material for: Bacterial Pathogens and Community Composition in Advanced Sewage Treatment Systems Revealed by Metagenomics Analysis Based on High-Throughput Sequencing
Source: PLoS One. 2015 May 4;10(5):e0125549. doi: 10.1371/journal.pone.0125549 (PMC4418606; doi:10.1371/journal.pone.0125549)
Supplement: S2 Table — (DOCX) [file pone.0125549.s002.docx]

**S2 Table.** Information of the water/sludge sampling from the sewage treatment plant and accession numbers of the sequencing datasets deposited into publicly available databases.

| **Sample ID** | **Sample Type** | **Sampling Time** | **MG-RAST ID** | **NCBI SRA ID** |
| --- | --- | --- | --- | --- |
| SI-1 | influent | 11/2012 | 4545779.3  4545877.3  4545878.3  4545879.3 | SRR1103268 |
| SI-2 |  | 02/2013 |  |  |
| SI-3 |  | 05/2013 |  |  |
| PE-1 | effluent | 11/2012 | 4545873.3  4545874.3  4545875.3  4545876.3 | SRR1103269 |
| PE-2 |  | 02/2013 |  |  |
| PE-3 |  | 05/2013 |  |  |
| AS-1 | activated sludge | 11/2012 | 4547054.3  4547057.3  4547080.3  4547081.3 | SRR1060401 |
| AS-2 |  | 02/2013 |  |  |
| AS-3 |  | 05/2013 |  |  |
| SE-1 | effluent | 11/2012 | 4545897.3  4545898.3  4545899.3  4545900.3 | SRR1103270 |
| SE-2 |  | 02/2013 |  |  |
| SE-3 |  | 05/2013 |  |  |
| FFE-1 | effluent | 11/2012 | 4546809.3  4546810.3  4546811.3  4545974.3 | SRR1103271 |
| FFE-2 |  | 02/2013 |  |  |
| FFE-3 |  | 05/2013 |  |  |
| FRE-1 | effluent | 11/2012 | 4545924.3  4545925.3  4545926.3  4545927.3 | SRR1103272 |
| FRE-2 |  | 02/2013 |  |  |
| FRE-3 |  | 05/2013 |  |  |
