# Supplementary material for: Bacterial Pathogens and Community Composition in Advanced Sewage Treatment Systems Revealed by Metagenomics Analysis Based on High-Throughput Sequencing
Source: PLoS One. 2015 May 4;10(5):e0125549. doi: 10.1371/journal.pone.0125549 (PMC4418606; doi:10.1371/journal.pone.0125549)
Supplement: S3 Table — (DOCX) [file pone.0125549.s003.docx]

**S3 Table.** Sequencing information of Illumina sequencing and 454 pyrosequencing data analyzed. In order to compare the samples at the same sequencing depth, the numbers of the Illumina and pyrosequencing reads for each sample were normalized to 9,000,000 and 6200, respectively.

| **Data ID** | **Illumina Sequencing** | | **454 Pyrosequencing** | |
| --- | --- | --- | --- | --- |
|  | **Raw** | **Denoised** | **Raw** | **Denoised** |
| SI | 16,880,074 | 13,355,979 | 10,473 | 6,934 |
| PI | 27,169,458 | 21,970,508 | 9,587 | 6,210 |
| AS | 13,993,784 | 9,087,773 | 13,371 | 10,773 |
| SE | 18,916,952 | 14,564,834 | 11,870 | 8,966 |
| FFE | 20,871,702 | 15,646,345 | 11,186 | 8,505 |
| FRE | 17,804,826 | 13,344,601 | 12,767 | 10,155 |
