# Supplementary material for: Bacterial Pathogens and Community Composition in Advanced Sewage Treatment Systems Revealed by Metagenomics Analysis Based on High-Throughput Sequencing
Source: PLoS One. 2015 May 4;10(5):e0125549. doi: 10.1371/journal.pone.0125549 (PMC4418606; doi:10.1371/journal.pone.0125549)
Supplement: S4 Table — (DOCX) [file pone.0125549.s004.docx]

**S4 Table.** Primer sequences and conditions for q-PCR of the target genes indicating human bacterial pathogens.

| Bacteria | Gene | Primer sequence (5’-3’) | Amplicon size (bp) | Cycling parameters | NCBI GenBank ID |
| --- | --- | --- | --- | --- | --- |
| *E.coli* | *uidA* | CAATGGTGATGTCAGCGTT(F)  ACACTCTGTCCGGCTTTTG(R) | 163 | 6 s at 95℃  8 s at 58℃  8 s at 72℃ | KP284853 |
| 1. *hydrophila* | *lip* | AACCTGGTTCCGCTCAAGCCGTTG(F)  TTGCTCGCCTCGGCCCAGCAGCT(R) | 760 | 60 s at 94℃  60 s at 62℃  90 s at 72℃ | KP284854 |
| 1. *butzleri* | *gyrA* | ATCTTTAGTATTCTTTACAAGAAATGG (F)  AACTGTTGTTCGTTTTCCA (R) | 308 | 30 s at 95℃  30 s at 60℃  60 s at 72℃ | KP284855 |
| *K. pneumoniae* | *phoE* | TGCCCAGACCGATAACTTTA(F)  CTGTTTCTTCGCTTCACGG(R) | 142 | 30 s at 95℃  30 s at 55℃  60 s at 72℃ | KP284856 |
