# Supplementary material for: Bacterial Pathogens and Community Composition in Advanced Sewage Treatment Systems Revealed by Metagenomics Analysis Based on High-Throughput Sequencing
Source: PLoS One. 2015 May 4;10(5):e0125549. doi: 10.1371/journal.pone.0125549 (PMC4418606; doi:10.1371/journal.pone.0125549)
Supplement: S5 Table — (DOCX) [file pone.0125549.s005.docx]

**S5** **Table.** Numbers of the sequences assigned to genera containing potentially pathogenic species obtained from annotation of 454 pyrosequencing reads by using MEGAN.

| Pathogenic Genus | SI | PE | AS | SE | FFE | FRE |
| --- | --- | --- | --- | --- | --- | --- |
| *Aeromonas* | 70 | 56 | 4 | 13 | 15 | 2 |
| *Arcobacter* | 3289 | 3781 | 72 | 33 | 40 | 8 |
| *Bacillus* | 0 | 0 | 0 | 0 | 0 | 1 |
| *Campylobacter* | 0 | 1 | 0 | 0 | 0 | 0 |
| *Clostridium* | 15 | 7 | 5 | 6 | 6 | 1 |
| *Corynebacterium* | 0 | 0 | 1 | 2 | 0 | 0 |
| *Enterobacter* | 3 | 2 | 2 | 0 | 0 | 0 |
| *Enterococcus* | 11 | 10 | 4 | 1 | 0 | 0 |
| *Helicobacter* | 0 | 0 | 0 | 0 | 1 | 0 |
| *Klebsiella* | 4 | 10 | 0 | 0 | 0 | 0 |
| *Legionella* | 0 | 0 | 1 | 2 | 0 | 22 |
| *Leptospira* | 0 | 0 | 2 | 0 | 0 | 0 |
| *Mycobacterium* | 0 | 0 | 3 | 5 | 9 | 15 |
| *Neisseria* | 0 | 0 | 0 | 0 | 0 | 1 |
| *Pseudomonas* | 20 | 5 | 1 | 9 | 8 | 6 |
| *Rickettsia* | 0 | 0 | 6 | 2 | 1 | 0 |
| *Serratia* | 1 | 1 | 1 | 0 | 0 | 0 |
| *Staphylococcus* | 1 | 0 | 0 | 0 | 0 | 0 |
| *Streptococcus* | 12 | 5 | 0 | 3 | 1 | 0 |
| *Treponema* | 2 | 5 | 0 | 0 | 2 | 0 |
| Total pathogenic sequences | 3428 | 3883 | 102 | 76 | 83 | 56 |
