# Supplementary material for: Bacterial Pathogens and Community Composition in Advanced Sewage Treatment Systems Revealed by Metagenomics Analysis Based on High-Throughput Sequencing
Source: PLoS One. 2015 May 4;10(5):e0125549. doi: 10.1371/journal.pone.0125549 (PMC4418606; doi:10.1371/journal.pone.0125549)
Supplement: S6 Table — (DOCX) [file pone.0125549.s006.docx]

**S6 Table.** Absolute abundance of genera containing potentially pathogenic species and virulence factors detected in sewage samples.

| Sample ID | | | | SI | PE | SE | FFE | FRE |
| --- | --- | --- | --- | --- | --- | --- | --- | --- |
| DNA Amount Per Volume (mean, ng/mL) | | | | 156.23 | 151.81 | 25.96 | 18.13 | 16.23 |
| DNA Amount Per Volume (SD, ng/mL) | | | | 11.91 | 9.17 | 4.23 | 3.74 | 3.53 |
| Sequence Number Per Milliliter Water | Genera Containing Potentially Pathogenic Species | 454 Pyrosequencing | *Aeromonas* | 547 | 425 | 17 | 14 | 2 |
|  |  |  | *Arcobacter* | 2.57×10^4^ | 2.87×10^4^ | 43 | 36 | 6 |
|  |  |  | *Clostridium* | 117 | 53 | 8 | 5 | 1 |
|  |  |  | *Enterobacter* | 23 | 15 | 0 | 0 | 0 |
|  |  |  | *Enterococcus* | 86 | 76 | 1 | 0 | 0 |
|  |  |  | *Klebsiella* | 31 | 76 | 0 | 0 | 0 |
|  |  |  | *Legionella* | 0 | 0 | 3 | 0 | 18 |
|  |  |  | *Mycobacterium* | 0 | 0 | 7 | 8 | 12 |
|  |  |  | *Pseudomonas* | 156 | 38 | 12 | 7 | 5 |
|  |  |  | *Streptococcus* | 94 | 38 | 4 | 1 | 0 |
|  |  |  | *Treponema* | 16 | 38 | 0 | 2 | 0 |
|  |  |  | Rare Genera | 16 | 15 | 5 | 2 | 2 |
|  |  |  | Total Abundance | 2.68×10^4^ | 2.95×10^4^ | 100 | 75 | 46 |
|  |  | Illumina Sequencing | *Aeromonas* | 116 | 103 | 0 | 0 | 0 |
|  |  |  | *Arcobacter* | 218 | 496 | 0 | 0 | 0 |
|  |  |  | *Bacillus* | 22 | 6 | 0 | 0 | 0 |
|  |  |  | *Enterobacter* | 15 | 10 | 0 | 0 | 0 |
|  |  |  | *Klebsiella* | 15 | 15 | 0 | 0 | 0 |
|  |  |  | *Mycoplasma* | 49 | 37 | 2 | 1 | 1 |
|  |  |  | *Neisseria* | 31 | 27 | 2 | 1 | 1 |
|  |  |  | *Pseudomonas* | 17 | 21 | 0 | 0 | 0 |
|  |  |  | *Streptococcus* | 15 | 14 | 0 | 0 | 0 |
|  |  |  | Rare Genera | 46 | 36 | 1 | 0 | 0 |
|  |  |  | Total Abundance | 544 | 765 | 5 | 2 | 2 |
|  | VFs | | Pathogenicity Islands | 56 | 39 | 0 | 0 | 0 |
|  |  |  | Virulence Proteins | 16 | 13 | 0 | 0 | 0 |

1. Rare Genera: Taxa with maximum abundance lower than 10 reads per milliliter water in any sample.
2. Absolute abundance was calculated by normalizing the sequence number of the potential pathogens or VFs in one nanogram of DNA to the amount of DNA extracted from the given volume of sewage samples (ng/mL).
