# Supplementary material for: Bacterial Pathogens and Community Composition in Advanced Sewage Treatment Systems Revealed by Metagenomics Analysis Based on High-Throughput Sequencing
Source: PLoS One. 2015 May 4;10(5):e0125549. doi: 10.1371/journal.pone.0125549 (PMC4418606; doi:10.1371/journal.pone.0125549)
Supplement: S7 Table — (DOCX) [file pone.0125549.s007.docx]

**S7 Table.** Numbers of the sequences assigned to genera containing potentially pathogenic species obtained from annotation of Illumina high-throughput sequencing reads by using MEGAN.

| Pathogenic Genus | SI | PE | AS | SE | FFE | FRE |
| --- | --- | --- | --- | --- | --- | --- |
| *Aeromonas* | 1485 | 1355 | 6 | 22 | 4 | 6 |
| *Arcobacter* | 2795 | 6533 | 19 | 25 | 24 | 4 |
| *Bacillus* | 283 | 72 | 25 | 19 | 4 | 2 |
| *Bordetella* | 0 | 1 | 0 | 0 | 1 | 0 |
| *Brucella* | 0 | 0 | 2 | 1 | 0 | 0 |
| *Campylobacter* | 39 | 72 | 0 | 0 | 1 | 0 |
| *Chlamydia* | 0 | 0 | 1 | 0 | 0 | 3 |
| *Clostridium* | 73 | 50 | 18 | 8 | 6 | 5 |
| *Corynebacterium* | 11 | 3 | 5 | 0 | 0 | 1 |
| *Enterobacter* | 191 | 141 | 1 | 4 | 0 | 0 |
| *Enterococcus* | 108 | 31 | 5 | 0 | 0 | 0 |
| *Escherichia* | 60 | 50 | 0 | 1 | 0 | 0 |
| *Francisella* | 7 | 11 | 15 | 18 | 5 | 9 |
| *Haemophilus* | 0 | 2 | 0 | 1 | 0 | 0 |
| *Helicobacter* | 32 | 47 | 3 | 2 | 0 | 1 |
| *Klebsiella* | 186 | 193 | 0 | 2 | 1 | 0 |
| *Legionella* | 1 | 0 | 3 | 11 | 6 | 4 |
| *Leptospira* | 1 | 0 | 9 | 1 | 0 | 1 |
| *Listeria* | 1 | 1 | 0 | 0 | 0 | 0 |
| *Mycobacterium* | 14 | 14 | 4 | 9 | 3 | 5 |
| *Mycoplasma* | 621 | 493 | 158 | 125 | 93 | 97 |
| *Neisseria* | 391 | 360 | 96 | 120 | 116 | 66 |
| *Pseudomonas* | 221 | 275 | 19 | 15 | 2 | 4 |
| *Rickettsia* | 0 | 0 | 1 | 10 | 5 | 0 |
| *Salmonella* | 1 | 4 | 0 | 0 | 0 | 0 |
| *Serratia* | 2 | 1 | 0 | 0 | 0 | 0 |
| *Shigella* | 111 | 88 | 1 | 5 | 2 | 0 |
| *Staphylococcus* | 30 | 18 | 37 | 14 | 12 | 6 |
| *Streptococcus* | 197 | 188 | 11 | 2 | 4 | 1 |
| *Treponema* | 7 | 5 | 0 | 0 | 0 | 0 |
| *Vibrio* | 57 | 52 | 1 | 3 | 3 | 4 |
| *Yersinia* | 27 | 26 | 0 | 0 | 0 | 0 |
| Total pathogenic sequences | 6952 | 10086 | 440 | 418 | 292 | 219 |
