# Supplementary material for: Bacterial Pathogens and Community Composition in Advanced Sewage Treatment Systems Revealed by Metagenomics Analysis Based on High-Throughput Sequencing
Source: PLoS One. 2015 May 4;10(5):e0125549. doi: 10.1371/journal.pone.0125549 (PMC4418606; doi:10.1371/journal.pone.0125549)
Supplement: S8 Table — (DOCX) [file pone.0125549.s008.docx]

**S8 Table.** Pathogenicity islands detected at six locations along sewage flow in the STP by Illumina high-throughput sequencing.

| MvirDB accession number | Name | Functions | Bacteria host | Number of reads | | | | | | Reference |
| --- | --- | --- | --- | --- | --- | --- | --- | --- | --- | --- |
|  |  |  |  | SI | PE | AS | SE | FFE | FRE |  |
| 16316 |  | secreted autotranspoter toxin | *Escherichia coli* | 1 | 2 |  |  |  |  | [[1](#_ENREF_1)] |
| 16321 | IucD | IucD protein | *Escherichia coli* | 3 | 5 |  |  |  |  | [[2](#_ENREF_2)] |
| 16324 | IucA | IucA protein | *Escherichia coli* | 3 | 1 |  |  |  |  | [[2](#_ENREF_2)] |
| 16415 | PapK | PapK protein | *Escherichia coli* | 1 |  |  |  |  |  | [[2](#_ENREF_2)] |
| 16417 | PapF | PapF protein | *Escherichia coli* | 1 | 1 |  |  |  |  | [[2](#_ENREF_2)] |
| 16423 |  | putative iron-regulated outer membrane virulence protein | *Escherichia coli* | 1 |  |  |  |  |  | [[3](#_ENREF_3)] |
| 16477 | PilV | PilV-like protein | *Escherichia coli* | 5 |  |  |  |  |  | [[4](#_ENREF_4)] |
| 16597 | YkfI-YafW | CP4-6 prophage; toxin of the YkfI-YafW toxin-antitoxin system | *Escherichia coli* | 3 |  |  |  |  |  | [[5](#_ENREF_5)] |
| 16599 |  | CP4-6 prophage; predicted DNA repair protein | *Escherichia coli* | 3 | 3 |  | 2 |  |  | [[5](#_ENREF_5)] |
| 16600 |  | CP4-6 prophage; predicted protein | *Escherichia coli* | 1 | 2 |  |  |  |  | [[5](#_ENREF_5)] |
| 16601 |  | CP4-6 prophage; predicted protein | *Escherichia coli* | 3 | 1 |  |  |  |  | [[5](#_ENREF_5)] |
| 16602 |  | CP4-6 prophage; predicted protein | *Escherichia coli* | 8 |  |  |  |  |  | [[5](#_ENREF_5)] |
| 16607 |  | CP4-6 prophage; partial regulator of insertion element IS911A | *Escherichia coli* | 4 | 7 |  |  |  |  | [[5](#_ENREF_5)] |
| 16609 |  | CP4-6 prophage; partial transposase of insertion element IS911A | *Escherichia coli* | 10 | 3 | 1 |  |  |  | [[5](#_ENREF_5)] |
| 16613 |  | CP4-6 prophage; S-methylmethionine:homocysteine methyltransferase | *Escherichia coli* | 5 | 8 |  |  |  |  | [[5](#_ENREF_5)] |
| 16614 |  | CP4-6 prophage; putative ferric transport protein ABC superfamily, atp_bind | *Escherichia coli* | 1 | 3 |  |  |  |  | [[5](#_ENREF_5)] |
| 16615 |  | CP4-6 prophage; predicted ferric trasnporter subunit | *Escherichia coli* | 9 | 2 |  |  |  |  | [[5](#_ENREF_5)] |
| 16619 |  | CP4-6 prophage; predicted DNA-binding transcriptional regulator | *Escherichia coli* | 1 | 7 |  | 1 |  |  | [[5](#_ENREF_5)] |
| 16620 |  | CP4-6 prophage; predicted lyase/synthase | *Escherichia coli* | 2 |  | 1 |  |  |  | [[5](#_ENREF_5)] |
| 16621 |  | CP4-6 prophage; predicted dehydratase | *Escherichia coli* | 3 | 4 |  |  |  |  | [[5](#_ENREF_5)] |
| 16622 |  | CP4-6 prophage; predicted sugar transporter | *Escherichia coli* | 1 |  |  |  |  |  | [[5](#_ENREF_5)] |
| 16623 |  | CP4-6 prophage; predicted xylosidase/arabinosidase | *Escherichia coli* | 2 | 2 |  |  |  |  | [[5](#_ENREF_5)] |
| 16624 |  | CP4-6 prophage; predicted DNA-binding transcriptional regulator | *Escherichia coli* | 18 |  |  |  |  |  | [[5](#_ENREF_5)] |
| 16625 |  | CP4-6 prophage; ornithine carbamoyltransferase 2, chain F | *Escherichia coli* | 2 | 17 |  |  |  |  | [[5](#_ENREF_5)] |
| 16628 |  | CP4-6 prophage; predicted protein | *Escherichia coli* | 1 | 3 |  |  |  |  | [[5](#_ENREF_5)] |
| 16633 |  | CP4-6 prophage; predicted phage integrase | *Escherichia coli* | 6 | 4 |  |  |  |  | [[5](#_ENREF_5)] |
| 16634 |  | DLP12 prophage; predicted integrase | *Escherichia coli* | 1 |  |  |  |  |  | [[6](#_ENREF_6)] |
| 16635 |  | DLP12 prophage; putative sensory transduction regulator | *Escherichia coli* | 1 |  |  |  |  |  | [[6](#_ENREF_6)] |
| 16636 |  | DLP12 prophage; predicted exonuclease | *Escherichia coli* | 2 |  |  | 1 |  |  | [[6](#_ENREF_6)] |
| 16642 | UPF0098 | DLP12 prophage; secreted protein, UPF0098 family | *Escherichia coli* | 4 | 1 |  |  |  |  | [[6](#_ENREF_6)] |
| 16643 |  | DLP12 prophage; predicted DNA-binding transcriptional regulator | *Escherichia coli* |  | 1 |  |  |  |  | [[6](#_ENREF_6)] |
| 16644 |  | DLP12 prophage; predicted protein | *Escherichia coli* |  | 1 |  |  |  |  | [[6](#_ENREF_6)] |
| 16645 |  | DLP12 prophage; conserved protein | *Escherichia coli* |  | 1 |  |  |  |  | [[6](#_ENREF_6)] |
| 16647 | RUS | DLP12 prophage; endonuclease RUS | *Escherichia coli* |  | 1 |  |  |  |  | [[6](#_ENREF_6)] |
| 16650 |  | DLP12 prophage; truncated outer membrane porin pseudogene | *Escherichia coli* | 3 | 2 |  |  |  |  | [[6](#_ENREF_6)] |
| 16651 |  | DLP12 prophage; predicted phage lysis protein | *Escherichia coli* |  | 4 |  |  |  |  | [[6](#_ENREF_6)] |
| 16652 |  | DLP12 prophage; predicted lysozyme | *Escherichia coli* | 1 | 3 |  |  |  |  | [[6](#_ENREF_6)] |
| 16655 |  | DLP12 prophage; putative envelope protein | *Escherichia coli* | 1 |  |  |  |  |  | [[6](#_ENREF_6)] |
| 16657 |  | DLP12 prophage; DNA packaging protein | *Escherichia coli* | 4 | 1 |  |  |  |  | [[6](#_ENREF_6)] |
| 16658 |  | DLP12 prophage; putative tail fiber assembly gene | *Escherichia coli* | 1 | 3 |  |  |  |  | [[6](#_ENREF_6)] |
| 16660 |  | DLP12 prophage; predicted protein | *Escherichia coli* | 2 |  |  |  |  |  | [[6](#_ENREF_6)] |
| 16662 |  | DLP12 prophage; outer membrane protease VII outer membrane protein 3b | *Escherichia coli* | 1 | 5 |  |  |  |  | [[6](#_ENREF_6)] |
| 16664 |  | CPS-53 KpLE1 prophage; bactoprenol-linked glucose translocase flippase | *Escherichia coli* |  | 1 |  |  |  |  | [[7](#_ENREF_7)] |
| 16668 |  | CPS-53 KpLE1 prophage; conserved protein | *Escherichia coli* |  | 2 |  |  |  |  | [[7](#_ENREF_7)] |
| 16671 |  | CPS-53 KpLE1 prophage; predicted protein | *Escherichia coli* | 7 | 3 |  | 1 |  |  | [[7](#_ENREF_7)] |
| 16672 |  | CPS-53 KpLE1 prophage; predicted defective phage replication protein O | *Escherichia coli* |  | 1 |  |  |  |  | [[7](#_ENREF_7)] |
| 16674 |  | CPS-53 KpLE1 prophage; predicted protein | *Escherichia coli* | 1 |  |  |  |  |  | [[7](#_ENREF_7)] |
| 16675 |  | CPS-53 KpLE1 prophage | *Escherichia coli* |  | 3 |  |  |  |  | [[7](#_ENREF_7)] |
| 16676 |  | CPS-53 KpLE1 prophage; predicted protein | *Escherichia coli* | 2 | 2 |  |  |  |  | [[7](#_ENREF_7)] |
| 16677 |  | CPS-53 KpLE1 prophage; predicted protein | *Escherichia coli* | 2 | 3 |  |  |  |  | [[7](#_ENREF_7)] |
| 16751 |  | putative peptidase encoded within prophage CP-933U | *Escherichia coli* |  | 1 |  |  |  |  | [[8](#_ENREF_8)] |
| 16947 |  | antitermination protein Q homolog of cryptic prophage CP-933M | *Escherichia coli* | 1 | 1 |  |  |  |  | [[8](#_ENREF_8)] |
| 17231 | AidA-I | AidA-I | *Escherichia coli* | 9 | 1 |  |  |  |  | [[9](#_ENREF_9)] |
| 17267 |  | bifunctional enterobactin receptor/adhesin protein | *Escherichia coli* | 13 | 6 |  |  |  |  | [[10](#_ENREF_10)] |
| 17271 | TerE | TerE | *Escherichia coli* |  | 1 |  |  |  |  | [[11](#_ENREF_11)] |
| 17272 | TerD | TerD | *Escherichia coli* |  | 11 |  |  |  |  | [[11](#_ENREF_11)] |
| 17273 | TerC | TerC | *Escherichia coli* | 2 | 10 |  |  |  |  | [[12](#_ENREF_12)] |
| 17274 | TerB | TerB | *Escherichia coli* |  | 1 |  |  |  |  | [[11](#_ENREF_11)] |
| 17275 | TerA | TerA | *Escherichia coli* |  | 3 |  |  |  |  | [[11](#_ENREF_11)] |
| 17302 | UreE | urease accessory protein UreE | *Escherichia coli* | 1 | 3 |  |  |  |  | [[13](#_ENREF_13)] |
| 17303 |  | urease subunit alpha | *Escherichia coli* |  | 2 | 1 |  |  |  | [[13](#_ENREF_13)] |
| 17305 |  | urease subunit gamma | *Escherichia coli* |  | 4 |  |  |  |  | [[13](#_ENREF_13)] |
| 17651 |  | prophage pi3 protein 58 | *Lactococcus lactis* |  | 1 |  |  |  |  | [[14](#_ENREF_14)] |
| 17659 |  | prophage pi3 protein 50 | *Lactococcus lactis* | 3 |  |  |  |  |  | [[14](#_ENREF_14)] |
| 17670 |  | prophage pi3 protein 40 | *Lactococcus lactis* | 2 |  |  |  |  |  | [[14](#_ENREF_14)] |
| 17671 |  | prophage pi3 protein 39 | *Lactococcus lactis* | 1 | 1 |  |  |  |  | [[14](#_ENREF_14)] |
| 17677 |  | prophage pi3 protein 33 | *Lactococcus lactis* | 1 |  |  |  |  |  | [[14](#_ENREF_14)] |
| 17689 |  | prophage pi3 protein 24 | *Lactococcus lactis* | 1 | 2 |  |  |  |  | [[14](#_ENREF_14)] |
| 17692 |  | prophage pi3 protein 21 | *Lactococcus lactis* | 6 |  |  |  |  |  | [[14](#_ENREF_14)] |
| 17698 |  | prophage pi3 protein 14 | *Lactococcus lactis* | 1 | 6 |  |  |  |  | [[14](#_ENREF_14)] |
| 17700 |  | prophage pi3 protein 12 | *Lactococcus lactis* | 8 |  |  | 1 |  |  | [[14](#_ENREF_14)] |
| 17701 |  | prophage pi3 protein 11 | *Lactococcus lactis* | 1 | 5 |  |  |  |  | [[14](#_ENREF_14)] |
| 17716 |  | prophage pi1 protein 07 | *Lactococcus lactis* |  | 1 |  |  |  |  | [[14](#_ENREF_14)] |
| 17717 |  | prophage pi1 protein 08 | *Lactococcus lactis* | 3 | 2 |  |  |  |  | [[14](#_ENREF_14)] |
| 17726 |  | prophage pi1 protein 15 | *Lactococcus lactis* | 1 | 1 |  |  |  |  | [[14](#_ENREF_14)] |
| 17745 |  | prophage pi1 protein 31 | *Lactococcus lactis* | 2 |  | 1 |  |  |  | [[14](#_ENREF_14)] |
| 17750 |  | prophage pi1 protein 35 | *Lactococcus lactis* | 2 |  |  |  |  |  | [[14](#_ENREF_14)] |
| 17755 |  | prophage pi1 protein 40 | *Lactococcus lactis* | 3 |  |  |  |  |  | [[14](#_ENREF_14)] |
| 17759 |  | prophage pi1 protein 44 | *Lactococcus lactis* | 1 |  |  |  |  |  | [[14](#_ENREF_14)] |
| 17760 |  | prophage pi1 protein 45 | *Lactococcus lactis* | 5 |  |  |  |  |  | [[14](#_ENREF_14)] |
| 17761 |  | prophage pi1 protein 46 | *Lactococcus lactis* | 1 |  |  |  |  |  | [[14](#_ENREF_14)] |
| 17801 |  | prophage Lp2 protein 21 | *Lactobacillus plantarum* |  | 2 |  |  |  |  | [[15](#_ENREF_15)] |
| 17804 |  | prophage Lp2 protein 24 | *Lactobacillus plantarum* |  | 1 |  |  |  |  | [[15](#_ENREF_15)] |
| 18653 |  | prophage LambdaSa1, antirepressor, putative | *Streptococcus agalactiae* |  | 1 |  |  |  |  | [[16](#_ENREF_16)] |
| 18683 |  | prophage LambdaSa1, structural protein, putative | *Streptococcus agalactiae* | 1 |  |  |  |  |  | [[16](#_ENREF_16)] |
| 18692 | pblA | prophage LambdaSa1, pblA protein, internal deletion | *Streptococcus agalactiae* | 3 | 1 |  |  |  |  | [[16](#_ENREF_16)] |
| 18712 | PblB | prophage LambdaSa2, PblB, putative | *Streptococcus agalactiae* | 1 | 1 |  |  |  |  | [[16](#_ENREF_16)] |
| 18725 |  | prophage LambdaSa2, terminase large subunit, putative | *Streptococcus agalactiae* | 2 |  |  |  |  |  | [[16](#_ENREF_16)] |
| 18729 |  | prophage LambdaSa2, site-specific recombinase phage integrase family protein | *Streptococcus agalactiae* | 6 |  |  |  |  |  | [[16](#_ENREF_16)] |
| 18808 |  | fructose-bisphosphate aldolase | *Streptococcus agalactiae* | 1 | 7 | 1 |  |  |  | [[17](#_ENREF_17)] |
| 19277 | IS629-2 | IS629 ORF2 | *Shigella flexneri* | 13 |  |  |  |  |  | [[18](#_ENREF_18)] |
| 19308 | IS911-2 | IS911 ORF2 | *Shigella flexneri* | 6 | 6 |  | 1 |  |  | [[19](#_ENREF_19)] |
| 19309 | IS911-2 | IS911 ORF2 | *Shigella flexneri* |  | 2 |  |  |  |  | [[19](#_ENREF_19)] |
| 19310 | IS911-1 | IS911 ORF1 | *Shigella flexneri* | 1 | 3 |  |  |  |  | [[19](#_ENREF_19)] |
| 19323 | ISSfl4-3 | ISSfl4 ORF3 | *Shigella flexneri* | 5 |  |  |  |  |  | [[20](#_ENREF_20)] |
| 19333 | IS600-2 | IS600 ORF2 | *Shigella flexneri* | 27 | 5 |  |  |  |  | [[21](#_ENREF_21)] |
| 19335 | IS10R | iso-IS10R ORF | *Shigella flexneri* | 1 | 13 |  |  |  |  | [[18](#_ENREF_18)] |
| 19355 | IS629-A | IS629 orfA | *Shigella flexneri* |  | 2 |  |  |  |  | [[20](#_ENREF_20)] |
| 19392 | ISSfl3-B | ISSfl3 orfB | *Shigella flexneri* |  | 1 |  |  |  |  | [[20](#_ENREF_20)] |
| 19393 | IS600-A | IS600 orfA | *Shigella flexneri* | 2 | 1 |  |  |  |  | [[21](#_ENREF_21)] |
| 19394 | IS600-B | IS600 orfB | *Shigella flexneri* | 36 | 1 |  | 1 |  |  | [[21](#_ENREF_21)] |
| 19400 | IS1-B | IS1 orfB | *Shigella flexneri* | 13 | 26 | 1 |  |  |  | [[20](#_ENREF_20)] |
| 19401 | IS1-A | IS1 orfA | *Shigella flexneri* | 81 | 11 |  | 1 |  |  | [[20](#_ENREF_20)] |
| 19707 |  | insertion element IS1 protein | *Salmonella enterica* | 208 | 73 |  | 3 |  |  | [[22](#_ENREF_22)] |
| 19708 | InsB | insertion element IS1 protein InsB | *Salmonella enterica* | 1 | 148 | 2 | 2 |  |  | [[22](#_ENREF_22)] |
| 20219 |  | phage DNA binding protein | *Salmonella enterica* | 1 |  |  |  |  |  | [[23](#_ENREF_23)] |
| 20220 |  | phage immunity repressor protein | *Salmonella enterica* | 1 |  |  |  |  |  | [[23](#_ENREF_23)] |
| 20222 |  | P4 phage protein | *Salmonella enterica* |  | 1 |  |  |  |  | [[24](#_ENREF_24)] |
| 20388 |  | Fels-2 prophage: probable prophage lysozyme | *Salmonella typhimurium* | 21 |  |  |  |  |  | [[25](#_ENREF_25)] |
| 20419 | PhoB | PhoB-dependent, ATP-binding pho regulon component | *Salmonella typhimurium* | 12 |  | 1 |  |  |  | [[26](#_ENREF_26)] |
| 20479 |  | paraquat-inducible protein B | *Salmonella enterica* | 9 | 10 | 1 |  |  |  | [[27](#_ENREF_27)] |
| 20480 |  | paraquat-inducible protein A | *Salmonella enterica* | 1 | 7 |  |  |  |  | [[27](#_ENREF_27)] |
| 20534 | STM1008 | Gifsy-2 prophage | *Salmonella typhimurium* | 3 | 5 |  | 3 | 7 | 5 | [[28](#_ENREF_28)] |
| 20575 |  | anaerobic dimethyl sulfoxide reductase subunit C | *Salmonella enterica* | 7 |  |  |  |  |  | [[29](#_ENREF_29)] |
| 20576 |  | anaerobic dimethyl sulfoxide reductase subunit B | *Salmonella enterica* | 45 |  |  |  |  |  | [[29](#_ENREF_29)] |
| 20577 |  | anaerobic dimethyl sulfoxide reductase subunit A | *Salmonella enterica* | 1 |  |  |  |  |  | [[29](#_ENREF_29)] |
| 20998 | YbtT | yersiniabactin biosynthetic protein YbtT | *Yersinia pestis* | 1 |  |  |  |  |  | [[30](#_ENREF_30)] |
| 21018 |  | yersiniabactin thioesterase | *Yersinia pestis* | 6 |  |  |  |  |  | [[30](#_ENREF_30)] |
| 20154 |  | bacteriophage recombination protein | *Salmonella enterica* | 1 | 1 |  | 1 | 1 | 1 | [[31](#_ENREF_31)] |
| 20126 |  | putative prophage terminase large subunit | *Salmonella enterica* |  | 2 |  |  | 1 |  | [[31](#_ENREF_31)] |

Reference:

1. Guyer D.M., Radulovic S., Jones F.E., Mobley H.L. (2002) Sat, the secreted autotransporter toxin of uropathogenic *Escherichia coli*, is a vacuolating cytotoxin for bladder and kidney epithelial cells. Infection and Immunity. 70(8), 4539-4546.

2. Kanamaru S., Kurazono H., Nakano M., Terai A., Ogawa O., et al. (2006) Subtyping of uropathogenic *Escherichia coli* according to the pathogenicity island encoding uropathogenic‐specific protein: Comparison with phylogenetic groups. International Journal of Urology. 13(6), 754-760.

3. Curtis N., Eisenstadt R.L., East S.J., Cornford R.J., Walker L.A., et al. (1988) Iron-regulated outer membrane proteins of *Escherichia coli* K-12 and mechanism of action of catechol-substituted cephalosporins. Antimicrobial Agents and Chemotherapy. 32(12), 1879-1886.

4. Pizarro-Cerda J., Cossart P. (2006) Bacterial adhesion and entry into host cells. Cell. 124(4), 715-727.

5. Fortier L.C., Sekulovic O. (2013) Importance of prophages to evolution and virulence of bacterial pathogens. Virulence. 4(5), 354-365.

6. Lindsey D., Mullin D., Walker J. (1989) Characterization of the cryptic lambdoid prophage DLP12 of *Escherichia coli* and overlap of the DLP12 integrase gene with the tRNA gene argU. Journal of Bacteriology. 171(11), 6197-6205.

7. Panis G., Duverger Y., Champ S., Ansaldi M. (2010) Protein binding sites involved in the assembly of the KplE1 prophage intasome. Virology. 404(1), 41-50.

8. Garmendia J., Phillips A.D., Carlier M.F., Chong Y., Schüller S., et al. (2004) TccP is an enterohaemorrhagic *Escherichia coli* O157: H7 type III effector protein that couples Tir to the actin‐cytoskeleton†. Cellular Microbiology. 6(12), 1167-83.

9. Benz I., Schmidt M.A. (1989) Cloning and expression of an adhesin (AIDA-I) involved in diffuse adherence of enteropathogenic *Escherichia coli*. Infection and Immunity. 57(5), 1506-1511.

10. Kaper J.B., Nataro J.P., Mobley H.L. (2004) Pathogenic *Escherichia coli*. Nature Reviews Microbiology. 2(2), 123-140.

11. Mitchell J.R., Wood E., Collins K. (1999) A telomerase component is defective in the human disease dyskeratosis congenita. Nature. 402(6761), 551-555.

12. Yamaguchi H., Baerlocher G.M., Lansdorp P.M., Chanock S.J., Nunez O., et al. (2003) Mutations of the human telomerase RNA gene (TERC) in aplastic anemia and myelodysplastic syndrome. Blood. 102(3), 916-918.

13. Cox G.M., Mukherjee J., Cole G.T., Casadevall A., Perfect J.R. (2000) Urease as a virulence factor in experimental cryptococcosis. Infection and Immunity. 68(2), 443-448.

14. Garcia P., Ladero V., Suárez J. (2003) Analysis of the morphogenetic cluster and genome of the temperate *Lactobacillus casei* bacteriophage A2. Archives of Virology. 148(6), 1051-1070.

15. Ventura M., Canchaya C., Kleerebezem M., de Vos W.M., Siezen R.J., et al. (2003) The prophage sequences of *Lactobacillus plantarum* strain WCFS1. Virology. 316(2), 245-255.

16. Pritchard D.G., Dong S., Kirk M.C., Cartee R.T., Baker J.R. (2007) LambdaSa1 and LambdaSa2 prophage lysins of *Streptococcus agalactiae*. Applied and Environmental Microbiology. 73(22), 7150-7154.

17. Salvatore F., Izzo P., Paolella G. (1986) Aldolase gene and protein families: structure, expression and pathophysiology. Horizons in Biochemistry and Biophysics. 8, 611.

18. Jin Q., Yuan Z., Xu J., Wang Y., Shen Y., et al. (2002) Genome sequence of *Shigella flexneri* 2a: insights into pathogenicity through comparison with genomes of *Escherichia coli* K12 and O157. Nucleic Acids Research. 30(20), 4432-4441.

19. Prere M., Chandler M., Fayet O. (1990) Transposition in *Shigella dysenteriae*: isolation and analysis of IS911, a new member of the IS3 group of insertion sequences. Journal of Bacteriology. 172(7), 4090-4099.

20. Venkatesan M.M., Goldberg M.B., Rose D.J., Grotbeck E.J., Burland V., et al. (2001) Complete DNA sequence and analysis of the large virulence plasmid of *Shigella flexneri*. Infection and Immunity. 69(5), 3271-3285.

21. Buchrieser C., Glaser P., Rusniok C., Nedjari H., d'Hauteville H., et al. (2000) The virulence plasmid pWR100 and the repertoire of proteins secreted by the type III secretion apparatus of *Shigella flexneri*. Molecular Microbiology. 38(4), 760-771.

22. Olliver A., Valle M., Chaslus-Dancla E., Cloeckaert A. (2005) Overexpression of the multidrug efflux operon acrEF by insertional activation with IS1 or IS10 elements in *Salmonella enterica* serovar typhimurium DT204 acrB mutants selected with fluoroquinolones. Antimicrob Agents Chemother. 49(1), 289-301.

23. Waldor M.K., Friedman D.I., Adhya S.L. Phages: their role in bacterial pathogenesis and biotechnology: ASM Press; 2005.

24. Bishop A.L., Baker S., Jenks S., Fookes M., Gaora P.Ó., et al. (2005) Analysis of the hypervariable region of the *Salmonella enterica* genome associated with tRNAleuX. Journal of Bacteriology. 187(7), 2469-2482.

25. Figueroa‐Bossi N., Uzzau S., Maloriol D., Bossi L. (2001) Variable assortment of prophages provides a transferable repertoire of pathogenic determinants in *Salmonella*. Molecular Microbiology. 39(2), 260-272.

26. Fadl A., Galindo C., Sha J., Klimpel G., Popov V., et al. (2006) Global gene expression of a murein (Braun) lipoprotein mutant of *Salmonella enterica* serovar Typhimurium by microarray analysis. Gene. 374, 121-127.

27. Liang X., Pham X.Q., Olson M.V., Lory S. (2001) Identification of a genomic island present in the majority of pathogenic isolates of *Pseudomonas aeruginosa*. Journal of Bacteriology. 183(3), 843-853.

28. Figueroa‐Bossi N., Bossi L. (1999) Inducible prophages contribute to *Salmonella* virulence in mice. Molecular Microbiology. 33(1), 167-176.

29. Baltes N., Hennig-Pauka I., Jacobsen I., Gruber A.D., Gerlach G.F. (2003) Identification of dimethyl sulfoxide reductase in *Actinobacillus pleuropneumoniae* and its role in infection. Infection and Immunity. 71(12), 6784-6792.

30. Kunkle C.A., Schmitt M.P. (2003) Analysis of the *Corynebacterium diphtheriae* DtxR regulon: identification of a putative siderophore synthesis and transport system that is similar to the *Yersinia* high-pathogenicity island-encoded yersiniabactin synthesis and uptake system. Journal of Bacteriology. 185(23), 6826-6840.

31. Zhang S., Kingsley R.A., Santos R.L., Andrews-Polymenis H., Raffatellu M., et al. (2003) Molecular pathogenesis of *Salmonella enterica* serotype Typhimurium-induced diarrhea. Infection and Immunity. 71(1), 1-12.
