# Supplementary material for: Bacterial Pathogens and Community Composition in Advanced Sewage Treatment Systems Revealed by Metagenomics Analysis Based on High-Throughput Sequencing
Source: PLoS One. 2015 May 4;10(5):e0125549. doi: 10.1371/journal.pone.0125549 (PMC4418606; doi:10.1371/journal.pone.0125549)
Supplement: S9 Table — (DOCX) [file pone.0125549.s009.docx]

**S9 Table.** Virulence proteins detected at six locations along sewage treatment in the STP by Illumina high-throughput sequencing.

| MvirDB accession number | Name | Functions | Bacteria host | Number of Reads | | | | | | Reference |
| --- | --- | --- | --- | --- | --- | --- | --- | --- | --- | --- |
|  |  |  |  | SI | PE | AS | SE | FFE | FRE |  |
| 2443 |  | type IV pilus assembly protein | *Neisseria meningitidis* | 6 | 3 |  |  |  |  | [[1](#_ENREF_1)] |
| 2675 |  | phosphomannomutase | *Yersinia enterocolitica* | 2 |  |  |  |  |  | [[2](#_ENREF_2)] |
| 2482 | aceA | aceA | *Mycobacterium tuberculosis* |  |  |  | 2 |  |  | [[3](#_ENREF_3)] |
| 7196 |  | bacterioferritin, iron storage and detoxification protein | *Escherichia coli* | 1 | 2 |  |  |  |  | [[4](#_ENREF_4)] |
| 7208 |  | bacterioferrin | *Shigella flexneri* | 5 | 3 |  |  |  |  | [[5](#_ENREF_5)] |
| 7209 |  | bacterioferrin | *Salmonella typhimurium* | 12 | 6 |  |  |  |  | [[5](#_ENREF_5)] |
| 7210 |  | bacterioferritin-associated ferredoxin | *Salmonella typhimurium* | 2 |  |  |  |  |  | [[6](#_ENREF_6)] |
| 7256 | ExbB | ExbB | *Escherichia coli* | 1 |  |  |  |  |  | [[7](#_ENREF_7)] |
| 7316 |  | ferric enterobactin enterochelin binding protein | *Shigella flexneri* | 5 | 4 |  |  |  |  | [[8](#_ENREF_8)] |
| 7354 |  | ferritin | *Salmonella enterica* | 1 |  |  |  |  |  | [[9](#_ENREF_9)] |
| 7356 |  | cytoplasmic ferritin | *Shigella flexneri* | 2 | 1 |  |  |  |  | [[9](#_ENREF_9)] |
| 7357 |  | cytoplasmic ferritin | *Salmonella typhimurium* | 11 | 10 |  |  |  |  | [[9](#_ENREF_9)] |
| 7445 |  | KpLE2 phage-like element; citrate-dependent iron III transport protein ABC superfamily, peri_bind | *Escherichia coli* | 1 | 1 |  |  |  |  | [[10](#_ENREF_10)] |
| 7817 |  | extracytoplasmic-function sigma-70 factor | *Pseudomonas aeruginosa* | 1 | 1 |  |  |  |  | [[11](#_ENREF_11)] |
| 7827 |  | general secretion pathway protein E | *Pseudomonas aeruginosa* |  |  | 1 |  |  |  |  |
| 7931 |  | UDP-glucose pyrophosphorylase | *Streptococcus pyogenes* | 13 | 2 |  |  |  |  | [[12](#_ENREF_12)] |
| 7989 |  | UDP-galactopyranose mutase | *Enterococcus faecalis* | 8 |  |  |  |  |  | [[13](#_ENREF_13)] |
| 8011 | trwD | trwD protein | *Bartonella henselae* |  |  |  | 1 |  |  | [[14](#_ENREF_14)] |
| 8012 | trwE | trwE protein | *Bartonella henselae* |  |  |  | 1 |  |  | [[14](#_ENREF_14)] |
| 8397 | RpoS | stationary phase specific sigma factor RpoS | *Legionella pneumophila* | 6 | 10 |  |  |  |  | [[15](#_ENREF_15)] |
| 8409 | DotB | defect in organelle trafficking protein DotB | *Legionella pneumophila* |  |  |  |  | 2 |  | [[16](#_ENREF_16)] |
| 8412 | DotO | IcmB DotO | *Legionella pneumophila* |  |  |  |  | 2 | 1 | [[16](#_ENREF_16)] |
| 8420 | DotH | IcmK DotH | *Legionella pneumophila* |  |  |  | 1 |  |  | [[16](#_ENREF_16)] |
| 8421 | DotI | IcmL DotI | *Legionella pneumophila* |  |  | 1 | 1 |  |  | [[16](#_ENREF_16)] |
| 8424 | DotL | IcmO DotL | *Legionella pneumophila* |  |  |  | 1 | 2 | 1 | [[16](#_ENREF_16)] |
| 8483 | ureG | urease accessory protein ureG | *Helicobacter pylori* |  | 2 |  | 3 | 1 | 1 | [[17](#_ENREF_17)] |
| 8612 | DraC | DraC | *Escherichia coli* | 1 |  |  |  |  |  | [[18](#_ENREF_18)] |
| 8652 |  | putative cytotoxin | *Escherichia coli* |  | 1 |  |  |  |  | [[19](#_ENREF_19)] |
| 8664 | IbeB | IbeB | *Escherichia coli* | 7 | 5 |  |  |  |  | [[20](#_ENREF_20)] |
| 8666 | kpsD | kpsD | *Escherichia coli* | 4 | 5 |  |  |  |  | [[21](#_ENREF_21)] |
| 8670 | TraJ | TraJ | *Plasmid R1-19* | 3 |  |  |  |  |  | [[22](#_ENREF_22)] |
| 8714 | EspD | EspD | *Escherichia coli* | 1 |  |  |  |  |  | [[23](#_ENREF_23)] |
| 13333 |  | low calcium response protein D | *Yersinia pestis* | 4 | 1 |  |  |  |  | [[24](#_ENREF_24)] |
| 13372 |  | pyruvate kinase | *Salmonella typhimurium* | 51 | 47 |  |  |  |  | [[25](#_ENREF_25)] |
| 13454 |  | DNA mismatch repair protein | *Salmonella typhimurium* | 47 | 49 | 1 |  |  |  | [[26](#_ENREF_26)] |
| 13492 | IS629 | IS629 ORF1 | *Shigella flexneri* | 1 |  |  |  |  |  | [[27](#_ENREF_27)] |
| 13722 | FecD | FecD | *Shigella flexneri* | 1 | 3 |  |  |  |  | [[28](#_ENREF_28)] |
| 13726 | FecA | FecA | *Shigella flexneri* | 3 | 5 |  |  |  |  | [[28](#_ENREF_28)] |
| 13727 | FecR | FecR | *Shigella flexneri* | 1 | 1 |  |  |  |  | [[28](#_ENREF_28)] |
| 14048 | InsA | truncated InsA protein | *Escherichia coli* | 2 |  |  |  |  |  | [[29](#_ENREF_29)] |
| 14168 | ClpV1 | ClpV1 | *Pseudomonas aeruginosa* |  |  | 9 |  |  |  | [[30](#_ENREF_30)] |
| 14231 | PapB | PapB protein | *Escherichia coli* | 1 |  |  |  |  |  | [[31](#_ENREF_31)] |
| 26381 | Hsp70 | heat shock protein 70 | *Cryptosporidium parvum* |  | 1 |  | 4 |  |  | [[32](#_ENREF_32)] |
| 26468 | Hsp | organellar heat shock protein | *Eimeria tenella* | 4 | 2 | 1 | 1 | 2 | 1 | [[32](#_ENREF_32)] |

Reference:

1. Hahn H.P. (1997) The type-4 pilus is the major virulence-associated adhesin of *Pseudomonas aeruginosa* – a review. Gene. 192(1), 99-108.

2. Kim S.H., Ahn S.H., Lee J.H., Lee E.M., Kim N.H., et al. (2003) Genetic analysis of phosphomannomutase/phosphoglucomutase from *Vibrio furnissii* and characterization of its role in virulence. Archives of Microbiology. 180(4), 240-250.

3. McKinney J.D., zu Bentrup K.H., Muñoz-Elías E.J., Miczak A., Chen B., et al. (2000) Persistence of *Mycobacterium tuberculosis* in macrophages and mice requires the glyoxylate shunt enzyme isocitrate lyase. Nature. 406(6797), 735-738.

4. Chiancone E., Ceci P., Ilari A., Ribacchi F., Stefanini S. (2004) Iron and proteins for iron storage and detoxification. Biometals. 17(3), 197-202.

5. Reddy P.V., Puri R.V., Khera A., Tyagi A.K. (2012) Iron storage proteins are essential for the survival and pathogenesis of *Mycobacterium tuberculosis* in THP-1 macrophages and the guinea pig model of infection. Journal of Bacteriology. 194(3), 567-575.

6. Mey A.R., Wyckoff E.E., Kanukurthy V., Fisher C.R., Payne S.M. (2005) Iron and fur regulation in *Vibrio cholerae* and the role of fur in virulence. Infection and Immunity. 73(12), 8167-8178.

7. Pradel E., Guiso N., Menozzi F.D., Locht C. (2000) *Bordetella pertussis* TonB, a Bvg-independent virulence determinant. Infection and Immunity. 68(4), 1919-1927.

8. Yancey R.J., Breeding S., Lankford C. (1979) Enterochelin (enterobactin): virulence factor for *Salmonella typhimurium*. Infection and Immunity. 24(1), 174-180.

9. Velayudhan J., Castor M., Richardson A., Main‐Hester K.L., Fang F.C. (2007) The role of ferritins in the physiology of *Salmonella enterica* sv. Typhimurium: a unique role for ferritin B in iron‐sulphur cluster repair and virulence. Molecular Microbiology. 63(5), 1495-1507.

10. Hayashi T., Makino K., Ohnishi M., Kurokawa K., Ishii K., et al. (2001) Complete genome sequence of enterohemorrhagic *Eschelichia coli* O157: H7 and genomic comparison with a laboratory strain K-12. DNA Research. 8(1), 11-22.

11. Bashyam M.D., Hasnain S.E. (2004) The extracytoplasmic function sigma factors: role in bacterial pathogenesis. Infection, Genetics and Evolution. 4(4), 301-308.

12. Jayaswal R., Bressan R., Handa A. (1985) Effects of a mutation that eliminates UDP glucose-pyrophosphorylase on the pathogenicity of *Erwinia carotovora* subsp. carotovora. Journal of Bacteriology. 164(1), 473-476.

13. Kleczka B., Lamerz A.-C., van Zandbergen G., Wenzel A., Gerardy-Schahn R., et al. (2007) Targeted gene deletion of *Leishmania* major UDP-galactopyranose mutase leads to attenuated virulence. Journal of Biological Chemistry. 282(14), 10498-10505.

14. de Paz H.D., Sangari F.J., Bolland S., García-Lobo J.M., Dehio C., et al. (2005) Functional interactions between type IV secretion systems involved in DNA transfer and virulence. Microbiology. 151(11), 3505-3516.

15. Bachman M.A., Swanson M.S. (2001) RpoS co‐operates with other factors to induce *Legionella pneumophila* virulence in the stationary phase. Molecular Microbiology. 40(5), 1201-1214.

16. Shohdy N., Efe J.A., Emr S.D., Shuman H.A. (2005) Pathogen effector protein screening in yeast identifies *Legionella* factors that interfere with membrane trafficking. Proceedings of the National Academy of Sciences of the United States of America. 102(13), 4866-4871.

17. Zambelli B., Turano P., Musiani F., Neyroz P., Ciurli S. (2009) Zn^2+^‐linked dimerization of UreG from *Helicobacter pylori*, a chaperone involved in nickel trafficking and urease activation. Proteins: Structure, Function, and Bioinformatics. 74(1), 222-239.

18. Gospodarek E., Bogiel T., Zalas-Wiecek P. (2009) Communication between microorganisms as a basis for production of virulence factors. Polish Journal of Microbiology. 58(3), 191-198.

19. Rippere-Lampe K.E., O'Brien A.D., Conran R., Lockman H.A. (2001) Mutation of the gene encoding cytotoxic necrotizing factor type 1 (cnf 1) attenuates the virulence of uropathogenic *Escherichia coli*. Infection and Immunity. 69(6), 3954-3964.

20. Wang S., Shi Z., Xia Y., Li H., Kou Y., et al. (2012) IbeB is involved in the invasion and pathogenicity of avian pathogenic *Escherichia coli*. Veterinary Microbiology. 159(3), 411-419.

21. McNulty C., Thompson J., Barrett B., Lord L., Andersen C., et al. (2006) The cell surface expression of group 2 capsular polysaccharides in *Escherichia coli*: the role of KpsD, RhsA and a multi‐protein complex at the pole of the cell. Molecular Microbiology. 59(3), 907-922.

22. Camacho E.M., Casadesús J. (2005) Regulation of traJ transcription in the *Salmonella* virulence plasmid by strand‐specific DNA adenine hemimethylation. Molecular Microbiology. 57(6), 1700-1718.

23. Law D. (2000) Virulence factors of *Escherichia coli* O157 and other *Shiga* toxin‐producing *E. coli*. Journal of Applied Microbiology. 88(5), 729-745.

24. Price S., Cowan C., Perry R., Straley S. (1991) The *Yersinia pestis* V antigen is a regulatory protein necessary for Ca2 (+)-dependent growth and maximal expression of low-Ca^2+^ response virulence genes. Journal of Bacteriology. 173(8), 2649-2657.

25. Spellerberg B., Cundell D.R., Sandros J., Pearce B.J., Idänpään‐Heikkilä I., et al. (1996) Pyruvate oxidase, as a determinant of virulence in *Streptococcus pneumoniae*. Molecular Microbiology. 19(4), 803-813.

26. Heithoff D.M., Sinsheimer R.L., Low D.A., Mahan M.J. (1999) An essential role for DNA adenine methylation in bacterial virulence. Science. 284(5416), 967-970.

27. Burland V., Shao Y., Perna N.T., Plunkett G., Blattner F.R., et al. (1998) The complete DNA sequence and analysis of the large virulence plasmid of *Escherichia coli* O157: H7. Nucleic Acids Research. 26(18), 4196-4204.

28. Luck S.N., Turner S.A., Rajakumar K., Sakellaris H., Adler B. (2001) Ferric dicitrate transport system (Fec) of *Shigella flexneri* 2a YSH6000 is encoded on a novel pathogenicity island carrying multiple antibiotic resistance genes. Infection and Immunity. 69(10), 6012-21.

29. Dobrindt U., Blum-Oehler G., Nagy G., Schneider G., Johann A., et al. (2002) Genetic structure and distribution of four pathogenicity islands (PAI I536 to PAI IV536) of uropathogenic *Escherichia coli* strain 536. Infection and Immunity. 70(11), 6365-6372.

30. Mougous J.D., Cuff M.E., Raunser S., Shen A., Zhou M., et al. (2006) A virulence locus of *Pseudomonas aeruginosa* encodes a protein secretion apparatus. Science. 312(5779), 1526-1530.

31. Friedrich M.J., Kinsey N.E., Vila J., Kadner R.J. (1993) Nucleotide sequence of a 13.9 kb segment of the 90 kb virulence plasmid of *Salmonella typhimurium*: the presence of fimbriai biosynthetic genes. Molecular Microbiology. 8(3), 543-558.

32. Neckers L., Tatu U. (2008) Molecular chaperones in pathogen virulence: emerging new targets for therapy. Cell Host and Microbe. 4(6), 519-527.
