# Supplementary figures and images for: Bacterial Pathogens and Community Composition in Advanced Sewage Treatment Systems Revealed by Metagenomics Analysis Based on High-Throughput Sequencing
Source: PLoS One. 2015 May 4;10(5):e0125549. doi: 10.1371/journal.pone.0125549 (PMC4418606; doi:10.1371/journal.pone.0125549)

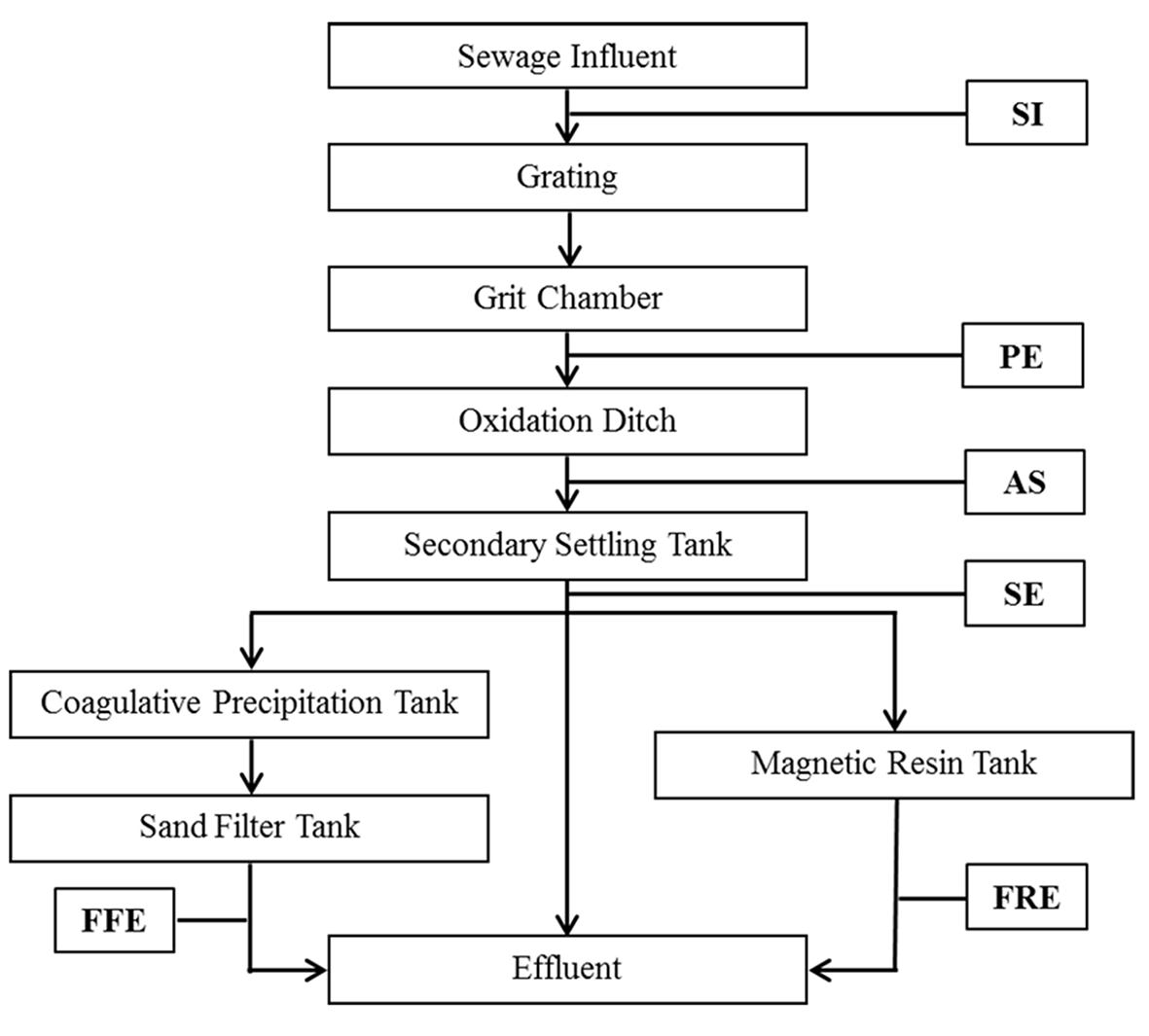

Supplement: S1 Fig — (TIF) [file pone.0125549.s010.tif]

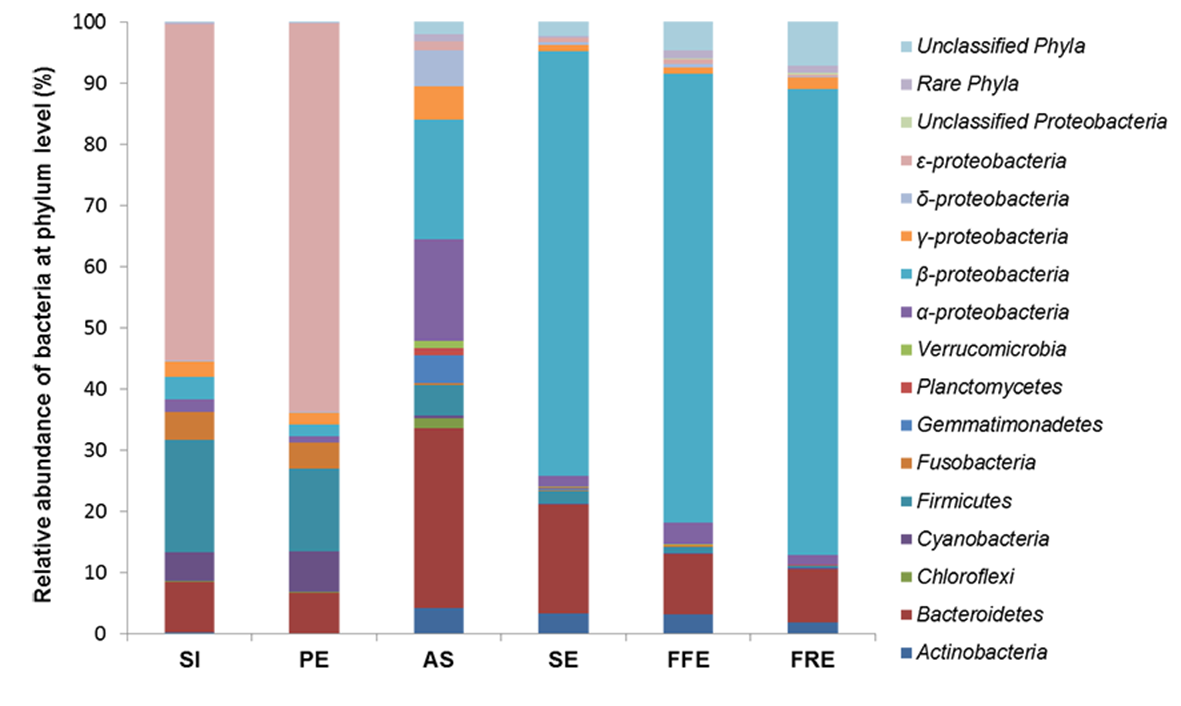

Supplement: S2 Fig — (TIF) [file pone.0125549.s011.tif]

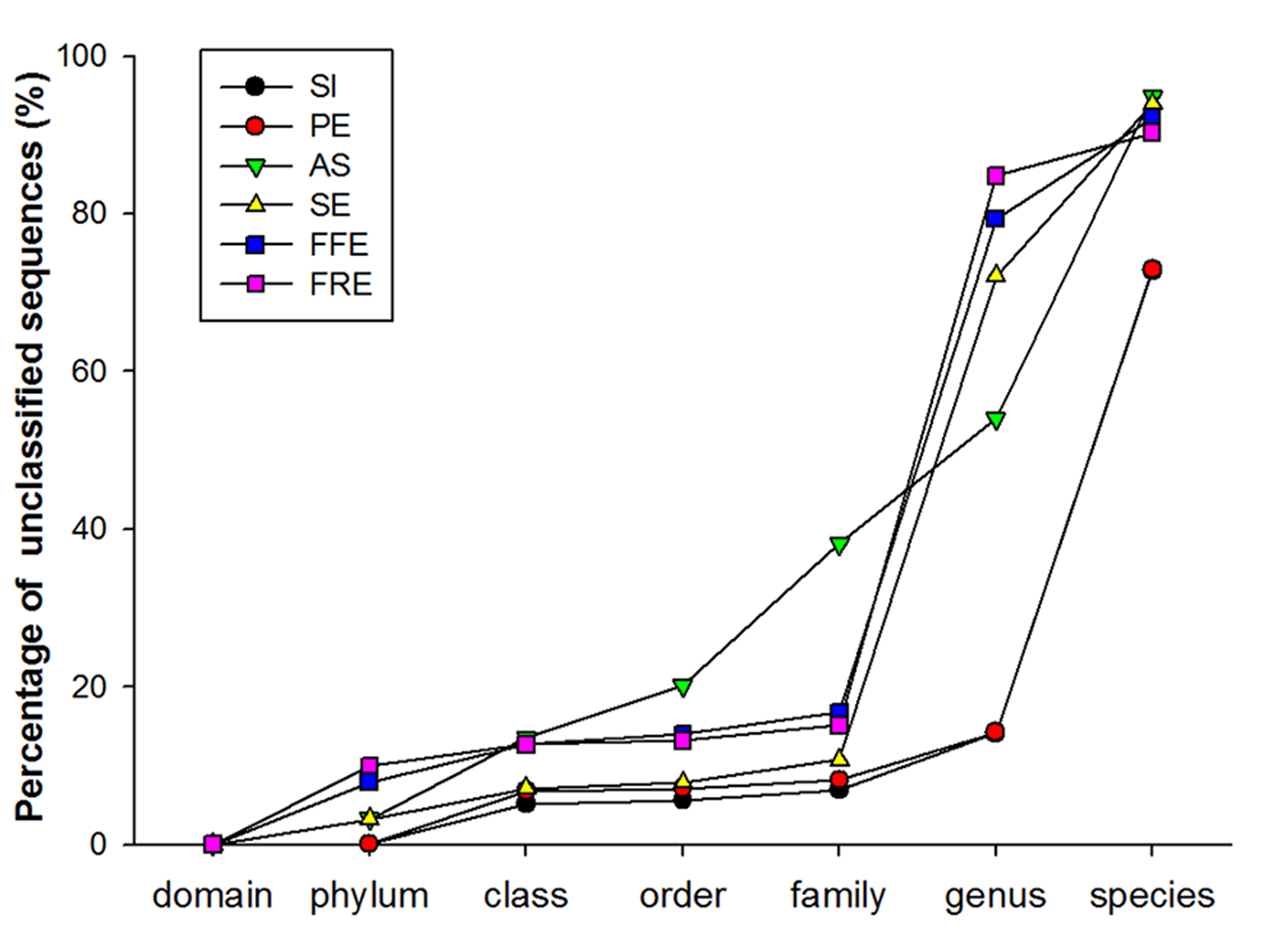

Supplement: S3 Fig — (TIF) [file pone.0125549.s012.tif]

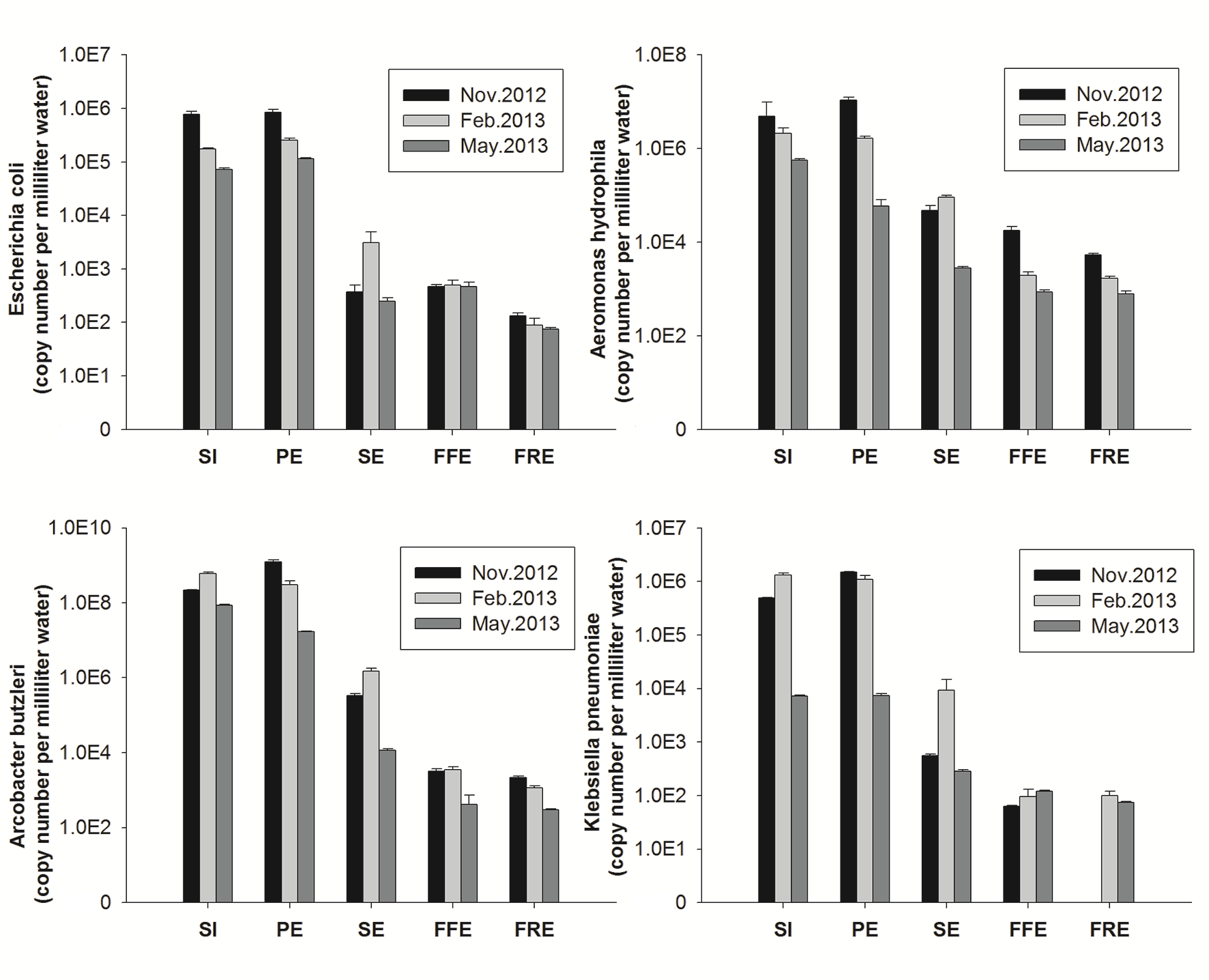

Supplement: S4 Fig — (TIF) [file pone.0125549.s013.tif]
